# Clinical efficacy of different therapeutic options for knee osteoarthritis: A network meta-analysis based on randomized clinical trials

**DOI:** 10.1371/journal.pone.0324864

**Published:** 2025-06-18

**Authors:** Xiao Chen, Yuanhe Fan, Hongliang Tu, Yuan Luo

**Affiliations:** 1 Department of Orthopedics, the First People’s Hospital of Neijiang, Neijiang, China; 2 Department of Rehabilitation, the First People’s Hospital of Neijiang, Neijiang, China; Government Law College, INDIA

## Abstract

**Objective:**

To assess and compare the clinical efficacy of various therapeutic options in treating patients with knee osteoarthritis (KOA).

**Methods:**

We performed a comprehensive search of PubMed, Embase, OVID, Cochrane Library and Web of Science databases from their inception to December 10th, 2023, identifying randomized controlled trials (RCTs) examining the effects of therapeutic options on KOA. Two researchers independently performed literature screening, data extraction, data collection and organization, and quality assessment. The data obtained were subjected to statistical analysis and graphical representation using Stata 17.0 software.

**Results:**

A total of 139 RCTs encompassing 9644 KOA patients and involving 12 therapeutic options were included. These interventions were low level laser therapy (LLLT), high intensity laser therapy (HILT), transcutaneous electrical nerve stimulation (TENS), interferential current (IFC), short wave diathermy, ultrasound, lateral wedged insole, knee brace, exercise, hydrotherapy, kinesio taping (KT) and extracorporeal shock wave therapy (ESWT). Regarding the WOMAC pain score, knee brace was determined to be the most likely to yield the best results, followed by exercise and HILT, ultrasound was worst intervention. In terms of WOMAC function score, knee brace emerged as the technique with the highest likelihood of being optimal, followed in sequence by hydrotherapy and ESWT, ultrasound was worst intervention. Knee brace ranked highest in effectiveness concerning the WOMAC stiffness score, followed by exercise and hydrotherapy. For the total WOMAC score, hydrotherapy demonstrated the highest probability of being the best technique, followed by exercise and HILT, short wave diathermy was worst intervention. In addressing VAS-rest, hydrotherapy exhibited the greatest likelihood of being the optimum technique, followed by HILT and LLLT. In terms of VAS-activity, knee brace had the highest probability of being the best technique, followed by LLLT and exercise, ultrasound was worst intervention. Overall, based on the results obtained from the SUCRA for all outcomes, knee brace had the highest probability of being the best technique, followed by hydrotherapy and exercise.

**Conclusion:**

The findings suggest that knee brace may be the most recommended therapeutic option for the knee osteoarthritis, followed by hydrotherapy and exercise.

## 1 Introduction

Knee osteoarthritis, a common chronic condition in the middle-aged and elderly, is clinically characterized by the degenerative changes within the knee joint. It presents with pain, stiffness, and functional limitations related to knee inflammation and effusion, potentially adversely impacting the quality of life [1]. Statistics indicate that more than 10% of individuals over the age of 60 are affected by this disease, which, along with its associated pain and functional disability, as well as the societal costs of joint replacement surgeries, represents a considerable burden [2].

Knee osteoarthritis (KOA) treatment strategies include a spectrum of options such as pharmacotherapy, interventional treatments, regenerative therapies involving cellular and acellular approaches, and joint replacement surgeries [3]. Nonsteroidal Anti-Inflammatory Drugs (NSAIDs) and acetaminophen are the most frequently prescribed analgesics for managing pain and enhancing physical function in osteoarthritis patients. Estimates indicate that between 10% to 35% of osteoarthritis patients utilize oral NSAIDs or acetaminophen for symptom management. However, the consumption of these medications is linked to gastrointestinal and cardiovascular adverse events and potentially an elevated risk of mortality, particularly among elderly patients with concurrent health issues [4,5].

Physical therapy offers a safer alternative to pharmacological interventions with a lower incidence of side effects. It is essential to investigate the efficacy of various physical therapy methods in the treatment of KOA. The array of physical therapy techniques includes low level laser therapy, high intensity laser therapy, transcutaneous electrical nerve stimulation, interferential current, short wave diathermy, ultrasound, lateral wedged insole, knee brace, exercise, hydrotherapy, kinesio taping and extracorporeal shock wave therapy. While existing research has primarily focused on comparing physical therapy with other treatment options, there is a paucity of studies that directly assess the comparative efficacy of different physical therapy approaches. This gap in the literature limits our ability to discern the relative advantages and disadvantages of each method in the context of KOA patient care [6].

Consequently, the objective of this study is to conduct a network meta-analysis to evaluate the clinical efficacy of the aforementioned physical therapy interventions for KOA. The findings aim to equip clinical practice with the valuable evidence-based medical evidence.

## 2 Method

### 2.1 Protocol and registration

This study adheres to the Preferred Reporting Items for Systematic Reviews and Meta-Analyses (PRISMA) 2020 statement, ensuring a structured methodology and reporting format, and A MeaSurement Tool to Assess systematic Reviews (AMSTAR) 2 guidelines. Furthermore, the network meta-analysis protocol has been duly registered on PROSPERO data base (the registration number: CRD42023473080).

### 2.2 Data sources

A comprehensive literature search was conducted by two independent researchers, with any disparities resolved through consultation with a third investigator. The search encompassed titles and abstracts, and full-text assessments were carried out as needed to determine study eligibility.

The following databases were systematically searched from their inception until December 10th, 2023: PubMed, Embase, OVID, Cochrane Library, Web of Science, and Scopus databases. Eligible studies encompassed only randomized controlled trials involving participants diagnosed with knee osteoarthritis. These studies compared various therapeutic options. The search utilized the following relevant terms: “osteoarthritis”, “knee”, “laser”, “transcutaneous electrical nerve stimulation”, “interferential current”, “short wave”, “ultrasound”, “wedged insole”, “brace”, “exercise”, “hydrotherapy”, “kinesio taping”, “extracorporeal shock wave therapy” and “random”. Additionally, Google Scholar was searched to identify potentially relevant literature. Furthermore, the reference lists of identified reports were meticulously reviewed to identify any additional pertinent studies. Only articles published in the English language were considered for inclusion in our network meta-analysis.

### 2.3 Eligibility criteria

The inclusion criteria were as follows: (1) Participants: Aged 18 years and above, diagnosed with unilateral or bilateral KOA based on the American College of Rheumatology criteria, categorized as mild-to-moderate KOA according to the Kellgren-Lawrence radiographic classification, and presented with knee pain. If both knees were affected, the knee with worse symptoms was included in the outcome assessment; (2) Types of Studies: Relevant randomized controlled trials (RCTs); (3) Types of Interventions: Studies comparing any combination of the 12 therapeutic options mentioned above and placebo were included. Each study needed to involve at least two of these therapies. (4)Types of Outcomes: Western Ontario and McMaster Universities Osteoarthritis Index (WOMAC) (pain, stiffness, function and total score) and visual analog scale (VAS) (rest and activity) at last follow-up.

The exclusion criteria were as follows: (1) non-RCTs, Case reports, review articles, meta-analyses, editorials, letters, animal studies, and cadaveric trials. (2) Patients with a previous history of knee surgery, decompensated organ failure, treatment for oncological diseases, systemic infammatory diseases, infectious diseases, intra-articular injection within the last 6 months. (3) Physical therapy or balneological treatment within the last 1 year, and change in the drug routine within last 2 months. (4) Studies with incomplete or missing data. (5) Duplicate articles. (6) Poor-quality research literature or studies lacking rigor in their design. (7) Papers with abstracts only and RCT protocols. (8) Non-English articles.

### 2.4 Data extraction

A specifically designed form was employed to extract essential information from each enrolled study. The data that was extracted included: (1) General information such as the lead author, year of publication, study design, country of study, study period, and follow-up time; (2) Demographic information, including the number and proportion of male or female patients, age at diagnosis, and the number of patients involved; (3) Details regarding the therapeutic options (intervention and comparison); (4) Information on clinical outcomes, including the pain, stiffness, function and total score of WOMAC and VAS (rest and activity) at last follow-up. In instances where SD was not available from the publication, SD was imputed using the method prescribed in the Cochrane Handbook.

### 2.5 Quality assessment

For RCTs, the Cochrane Risk of Bias Tool was employed to assess the quality. The risk of bias for the included trials was evaluated by two researchers (the first and second authors) based on the Cochrane Handbook criteria.

### 2.6 Statistical analysis

To conduct a comprehensive network meta-analysis, we utilized the statistical software packages “Network” and “mvmeta” within STATA 17.0 software. Continuous variables including WOMAC and VAS were analyzed using weighted mean differences (WMD) with corresponding 95% CI. When the 95% CI of the contained the value 1, the comparison was considered statistically non-significant.

For direct comparisons, a conventional meta-analysis was conducted to aggregate the results using random-effects models, serving as sensitivity analyses. The network meta-analysis employed a frequentist approach with a random-effects model to estimate both direct and indirect comparisons. The primary objective of the network meta-analysis was to assess whether any of the comparator interventions demonstrated superiority. To evaluate potential inconsistencies between indirect and direct comparisons, we employed global inconsistency, local inconsistency (using a node-splitting approach), and loop inconsistency. Statistical significance for global inconsistency was determined using P-values, with P > 0.05 indicating no significant global inconsistency. Local inconsistency was assessed through node-splitting analysis, and P > 0.05 indicated no significant local inconsistency. Heterogeneity within each closed loop was estimated using the inconsistency factor (IF), with a 95% CI (IF) value of zero signifying no statistical significance. In each pre-specified outcome, a global network diagram was employed to illustrate direct comparisons between interventions. The size of the nodes in the diagram corresponded to the number of participants receiving each treatment. Treatments subject to direct comparisons were linked by lines, and the thickness of these lines was proportional to the number of trials evaluating the specific comparison.

All intervention measures were ranked based on their respective SUCRA values or the area under the curve, resulting in a comprehensive ranking of the interventions. To assess the potential for publication bias, the comparison-adjusted funnel plot was utilized. This analysis aimed to determine whether there was evidence of a small sample effect or publication bias within the intervention network.

## 3 Results

### 3.1 Search results

**Fig 1:**
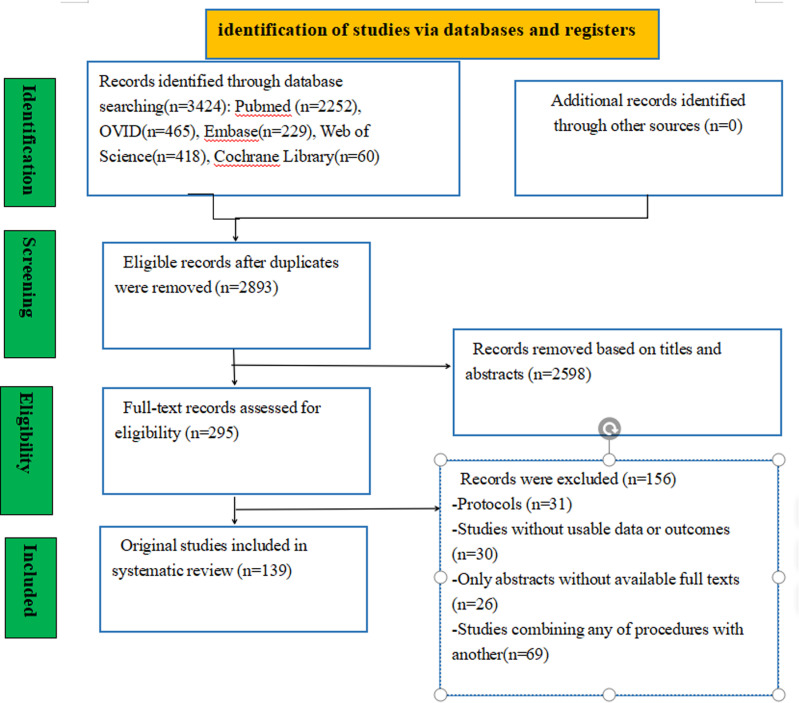
Flow chart of included studies.

A total of 3424 studies were initially identified from various sources, including PubMed (n = 2252), Embase (n = 229), Ovid (n = 465), Web of Science (n = 418), and the Cochrane Library (n = 60). After removing duplicate and irrelevant literatures, a full text search was conducted for the remaining 295 literatures. Ultimately, 139 studies, involving 9644 patients, met the eligibility criteria for inclusion in this network meta-analysis. Participants: This study included 9644 patients with KOA (age ≥ 18 years). And it involved 12 interventions: LLLT, HILT, TENS, IFC, short wave diathermy, ultrasound, lateral wedged insole, knee brace, exercise, hydrotherapy, KT, and ESWT. The control was a placebo. Outcomes included WOMAC pain, function, and stiffness scores at last follow-up, WOMAC total score, and VAS scores at rest and after activity. The process of study selection is illustrated in **Fig 1**, and the baseline characteristics of the included studies are summarized in **Table 1**.

**Table 1:** Baseline characteristics of the included studies.

–	Country	Study design	Intervention	N	Sex (M/F)	Age (years)	BMI	Follow-up	Outcome
Adıgüzel 2021	Turkey	RCT	Hydrotherapy	32	1/31	60.84 ± 7.76	30.34 ± 4.33	24 weeks	WOMAC, VAS
			Placebo	32	1/31	59.09 ± 9.15	31.25 ± 5.15		
Ahmad 2023	Malaysia	RCT	HILT	17	3/14	51.18 ± 9.79	30.58 ± 5.43	12 weeks	VAS
			LLLT	17	5/12	57.94 ± 10.56	27.57 ± 4.47		
Ahmad 2023	Malaysia	RCT	Exercise	40	19/21	65.27 ± 6.86	28.83 ± 3.38	8 weeks	VAS
			Placebo	40	15/25	65.60 ± 8.70	26.86 ± 4.37		
Akaltun 2021	Turkey	RCT	HILT	20	–	57.85 ± 8.06	29.93 ± 5.49	8 weeks	WOMAC, VAS
			Placebo	20	–	58.61 ± 11.28	31.95 ± 4.86		
Akyol 2010	Turkey	RCT	Short wave diathermy	20	0/20	57.8 ± 10.65	31.05 ± 5.22	12 weeks	WOMAC, VAS
			Placebo	20	0/20	56.6 ± 8.13	30.37 ± 3.25		
Alfredo 2020	Brazil	RCT	Ultrasound	20	6/14	64.35 ± 6.16	30.98 ± 3.36	8 weeks	WOMAC, VAS
			Placebo	20	6/14	62.7 ± 8.49)	31.09 ± 3.16		
Alfredo 2022	Brazil	RCT	LLLT	20	4/16	68.55 ± 9.62	28.39 ± 4.35	24 weeks	WOMAC
			Placebo	20	3/17	65.9 ± 8.82	29.16 ± 3.65		
Alghadir 2013	Saudi Arabia	RCT	LLLT	20	10/10	55.2 ± 8.14	32.34 ± 5.77	4 weeks	WOMAC, VAS
			Placebo	20	12/8	57 ± 7.77	33.09 ± 4.98		
Ali 2014	Pakistan	RCT	TENS	25	–	50.5 ± 4.6	–	4 weeks	WOMAC
			Placebo	25	–	50.5 ± 4.6	–		
Alireza 2023	Iran	RCT	Exercise	24	4/20	52.8 ± 9.6	29.22 ± 3.77	8 weeks	WOMAC, VAS
			Placebo	24	7/17	55.7 ± 9.2	31.3 ± 5.03		
Alkhawajah 2019	Arabia	RCT	Exercise	20	13/7	56.5 ± 7.6	32.6 ± 7.8	2 days	WOMAC
			Placebo	20	12/8	56.6 ± 8.5	33.3 ± 6.1		
Allen 2021	UA	RCT	Exercise	230	194/36	59.9 ± 9.9	33.9 ± 7.4	36 weeks	WOMAC
			Placebo	115	98/17	60.2 ± 11.1	33.9 ± 7.5		
Rashoud 2014	UK	RCT	LLLT	26	10/16	52 ± 9	38 ± 5.6	24 weeks	VAS
			Placebo	23	8/15	56 ± 11	37.1 ± 5.3		
Alqualo-Costa 2021	Brazil	RCT	IFC	42	13/29	64.5 ± 7.8	29.3 ± 5.2	24 weeks	WOMAC
			Placebo	42	14/28	65.3 ± 8.5	29.9 ± 4.6		
Amornthep 2023	Taiwan	RCT	LLLT	16	1/15	67.44 ± 6.54	–	1 week	WOMAC
			Placebo	16	5/11	71.63 ± 7.60	–		
Anandkumar 2014	India	RCT	KT	20	8/12	55.9 ± 5.0	–	–	VAS
			Placebo	20	9/11	55.7 ± 5.8	–		
Anwer 2014	India	RCT	Exercise	21	–	54.9 ± 7.7	26.5 ± 1.8	6 weeks	WOMAC
			Placebo	21	–	56.0 ± 6.8	27.1 ± 1.3		
Artuc 2023	Turkey	RCT	TENS	19	3/16	58.50 ± 9.80	30.33 ± 6.62	12 weeks	WOMAC, VAS
			IFC	18	4/14	61.95 ± 11.78	31.43 ± 3.51		
Assar 2020	Iran	RCT	Exercise	12	–	57.5 ± 6.9	28.5 ± 3.7	8 weeks	WOMAC, VAS
			Placebo	12	–	63.8 ± 7.5	23.1 ± 11.6		
Atamaz 2012	Turkey	RCT	TENS	37	6/31	61.9 ± 6.9	28.4 ± 3.5	24 weeks	WOMAC, VAS
			IFCs	31	4/21	62.0 ± 7.9	29.8 ± 3.4		
			Short wave diathermy	31	4/27	61.6 ± 7.4	28.5 ± 4.2		
Aydogdu 2017	Turkey	RCT	KT	28	–	52.53 ± 9.68	31.18 ± 5.14	12 weeks	WOMAC
			Placebo	26	–	51.19 ± 8.94	31.52 ± 5.70		
Baykal 2023	Turkey	RCT	KT	82	18/64	66.04 ± 6.36	–	–	VAS
			Placebo	82	20/62	64.98 ± 5.78	–		
Bennell 2015	Australia	RCT	Exercise	73	23/50	67.4 ± 8.6	29.3 ± 4.3	48 weeks	WOMAC, VAS
			Placebo	67	23/44	69.8 ± 7.5	28.9 ± 3.9		
Bennell 2011	Australia	RCT	Lateral wedge insole	103	41/62	63.3 ± 8.1	28.1 ± 4.2	52 weeks	WOMAC, VAS
			Placebo	97	31/56	65.0 ± 7.9	30.4 ± 5.6±		
Bruce-Brand 2012	Ireland	RCT	TENS	10	4/6	63.9 ± 5.8	33.7 ± 5.6	6 weeks	WOMAC
			Exercise	10	4/6	63.4 ± 5.9	33.9 ± 8.3		
			Placebo	6	3/3	65.2 ± 3.1	31.7 ± 4.1		
Cantista 2020	Spain	RCT	Hydrotherapy	60	–	–	–	12 weeks	WOMAC, VAS
			Placebo	60	–	–	–		
Carpenedo 2021	Italy	RCT	Ultrasound	8	2/6	70.37 ± 7.36	29.48 ± 4.42	24 weeks	VAS
			Placebo	8	3/5	70.87 ± 11.81	29.62 ± 3.43		
Chen 2014	China	RCT	Exercise	30	–	>40	–	24 weeks	WOMAC, VAS
			Ultrasound	30	–	>40	–		
			ESWT	30	–	>40	–		
			Placebo	30	–	>40	–		
Chen 2023	China	RCT	HILT	158	35/123	64.74 ± 6.38	24.98 ± 3.68	12 weeks	WOMAC
			Placebo	151	42/109	63.93 ± 5.86	24.64 ± 9.15		
Chen HX 2021	China	RCT	Exercise	10	3/7	59.63 ± 8.40	23.24 ± 0.85	6 weeks	WOMAC, VAS
			Placebo	8	2/6	58.30 ± 8.54	23.02 ± 1.37		
Chen PY 2021	China	RCT	Exercise	36	4/32	77.4 ± 5.9	24.7 ± 2.6	12 weeks	WOMAC, VAS
			Placebo	32	2/30	75.4 ± 6.4	24.0 ± 2.7		
Cherian 2016	USA	RCT	TENS	33	14/19	58 (33–77)	–	48 weeks	VAS
			Placebo	37	10/27	62 (27–86)	–		
Cho 2015	South Korea	RCT	KT	23	6/17	58.2 ± 4.5	–	–	VAS
			Placebo	23	7/16	57.5 ± 4.4	–		
Choi 2023	Korea	RCT	ESWT	9	4/5	73.7 ± 2.4	25.6 ± 2.9	4 weeks	WOMAC, VAS
			Placebo	9	5/4	72.6 ± 2.3	26.4 ± 2.0		
Danazumi 2021	Nigeria	RCT	KT	30	–	52.3 ± 5.19	24.1 ± 4.08	–	VAS
			Placebo	30	–	52.0 ± 6.25	23.9 ± 4.23		
Dantas 2023	Brazil	RCT	Exercise	16	7/9	60.9 ± 9.5	29.1 ± 3.5	8 weeks	WOMAC
			Placebo	14	8/6	63.1 ± 8.2	30.8 ± 3.9		
Dias 2016	Brazil.	RCT	Hydrotherapy	33	0/33	70.8 ± 5.00	30.5 ± 4.30	6 weeks	WOMAC, VAS
			Placebo	32	0/32	71.0 ± 5.20	30.0 ± 5.20		
Dogan 2022	Turkey	RCT	KT	27	0/27	56.9 ± 6.9	32.8 ± 5.8	8 weeks	VAS
			Placebo	30	0/30	55.7 ± 6.9	30.8 ± 5.4		
Donec 2020	Lithuanian	RCT	KT	81	17/64	68.7 ± 9.9	30.5 ± 5.3	4 weeks	VAS
			Placebo	76	16/60	70.6 ± 8.3	30.7 ± 5.2		
Ekici 2022	Turkey	RCT	HILT	30	13/17	61.07 ± 6.96	30.97 ± 3.31	12 weeks	WOMAC, VAS
			Placebo	26	7/19	57.85 ± 7.04	32.17 ± 4.79		
Elboim-Gabyzon 2023	Israel	RCT	TENS	20	7/13	62.7 ± 6.6	31.1 ± 3.3	3 weeks	WOMAC
			LLLT	20	5/15	63.0 ± 6.2	30.8 ± 3.7		
Fazli 2023	Iran	RCT	KT	28	17/11	55.7 ± 5.3	24.4 ± 3.04	4 weeks	WOMAC, VAS
			Placebo	28	11/17	54.4 ± 3.2	23.2 ± 2.7		
Fokmare 2023	India	RCT	Hydrotherapy	30	7/23	50.06 ± 6.66	–	2weeks	WOMAC, VAS
			Knee brace	30	9/21	51.43 ± 4.88	–		
Foley 2014	Australia	RCT	Hydrotherapy	35	20/15	73.0 ± 8.2	–	6 weeks	WOMAC
			Exercise	35	15/20	69.8 ± 9.2	–		
			Placebo	35	15/20	69.8 ± 9.0	–		
Foroughi 2011	Australia	RCT	Exercise	26	0/26	66 ± 8	31.4 ± 5.4	24 weeks	WOMAC
			Placebo	28	0/28	65 ± 7	32.7 ± 8.4		
Fukuda 2011	USA	RCT	Short wave diathermy	30	–	62.0 ± 8.0	29.4 ± 4.5	48 weeks	WOMAC, VAS
			Placebo	21	–	57.0 ± 9.0	27.6 ± 3.7		
Fukuda 2011	USA	RCT	LLLT	25	5/20	63.0 ± 9.0	30.0 ± 3.5	3 weeks	VAS
			Placebo	22	8/14	63.0 ± 8.0	28.7 ± 4.1		
Fung 2021	China	RCT	Exercise	37	3/34	75.1 ± 8.0	22.0 ± 2.0	–	WOMAC, VAS
			Placebo	36	2/34	76.1 ± 7.5	22.5 ± 2.2		
Gao 2023	China	RCT	Exercise	13	5/8	68.54 ± 2.07	26.38 ± 1.99	8 weeks	VAS
			Placebo	14	6/8	67.86 ± 1.41	26.47 ± 2.80		
Gholami 2023	UK	RCT	Exercise	32	–	–	–	24 weeks	WOMAC, VAS
			Placebo	32	–	–	–		
Gomes 2020	Brazil.	RCT	Placebo	20	2/18	69.4 ± 4.45	–	10 weeks	WOMAC
			TFC	20	2/18	71.85 ± 2.62	–		
			Short wave diathermy	20	1/19	68.45 ± 4.62	–		
			LLLT	20	0/20	65.75 ± 4.48	–		
Günaydin 2022	Turkey	RCT	KT	22	0/22	58.8 ± 6.2	28.8 ± 4.7	12 weeks	VAS
			ESWT	18	0/18	58.8 ± 6.2	28.8 ± 4.7		
			Placebo	20	0/20	58.8 ± 6.2	28.8 ± 4.7		
Guo 2021	China	RCT	Placebo	52	24/28	61.5 ± 7.2	27.6 ± 5.4	8 weeks	VAS
			Exercise	50	23/27	63.2 ± 8.0	27.4 ± 6.0		
Gur 2003	Turkey	RCT	LLLT	30	7/23	59.80 ± 8.03	28.49 ± 3.02	14 weeks	WOMAC, VAS
			Placebo	30	6/24	60.52 ± 6.91	30.27 ± 311		
Hammam 2020	Egypt	RCT	EWST	15	6/9	50.4 ± 3.4	30.7 ± 3.5	4 weeks	VAS
			Placebo	15	8/7	49.7 ± 3.1	31.1 ± 3		
Han 2021	China	RCT	Ultrasound	31	13/18	53.6 ± 19.1	–	24 weeks	WOMAC, VAS
			Placebo	31	14/17	55.6 ± 17.6	–		
Hinman 2003	Australia	RCT	KT	29	9/19	66 ± 8	29.3 ± 4.0	3 weeks	WOMAC, VAS
			Placebo	29	9/19	69 ± 9	30.1 ± 4.0		
Ho 2022	Taiwan	RCT	ESWT	18	6/12	65.6 ± 11	24.1 ± 2.4	3 weeks	WOMAC, VAS
			Placebo	18	5/13	64.6 ± 11.8	23.9 ± 1.4		
Hu 2019	China	RCT	Exercise	52	–	66.32 ± 4.16	36.49 ± 8.99	24 weeks	WOMAC, VAS
			Placebo	40	–	65.54 ± 3.59	26.4 ± 3.07		
Iijima 2020	Japan	RCT	TENS	30	7/23	59.9 ± 6.41	22.1 ± 2.94	–	VAS
			Placebo	30	10/20	58.2 ± 5.63	23.4 ± 4.03		
Imamura 2016	Brazil	RCT	ESWT	52	0/52	70.0 ± 6.5	–	12 weeks	WOMAC, VAS
			Placebo	53	0/53	72.4 ± 6.5)	–		
Itoh 2008	Japan	RCT	Placebo	6	–	62-83	–	10 weeks	WOMAC, VAS
			TENS	6	–	62-83	–		
Jang 2023	Korea	RCT	Short wave diathermy	53	1/52	61.45 ± 5.05	–	4 weeks	WOMAC, VAS
			Ultrasound	52	4/48	60.85 ± 5.11	–		
Jia 2022	China	RCT	Ultrasound	57	15/42	62.28 ± 10.88	25.18 ± 3.26	24 weeks	WOMAC
			Short wave diathermy	57	12/45	59.93 ± 8.97	25.29 ± 2.85		
Jones 2013	UK	RCT	Lateral wedged insole	28	–	66.3 ± 8.2	–	2 weeks	WOMAC, VAS
			Knee brace	28	–	66.3 ± 8.2	–		
Jorge 2023	Brazil	RCT	LLLT	44	18/26	59.1 ± 9.3	29.0 ± 3.4	24 weeks	WOMAC, VAS
			Placebo	42	16/26	58.4 ± 8.3	29.9 ± 3.4		
Karakas 2020	Turkey	RCT	Ultrasound	39	8/31	59.10 ± 7.45	28.70 ± 4.86	12 weeks	WOMAC, VAS
			Placebo	36	4/32	60.75 ± 7.46	29.22 ± 10.13		
Karimi 2021	Iran	RCT	Exercise	10	0/10	40.61 ± 8.54	–	8 weeks	WOMAC
			Placebo	10	0/10	65.6 ± 6.54	–		
Kayamutlu 2016	Turkey	RCT	KT	20	4/16	54.25 ± 6.01	30.72 ± 3.80	4 weeks	WOMAC, VAS
			Placebo	19	2/17	57.10 ± 6.26	31.34 ± 6.16		
Kayamutlu 2018	Turkey	RCT	Exercise	21	5/16	56.29 ± 6.64	30.74 ± 4.31	48 weeks	VAS
			TENS	22	3/19	57.77 ± 6.24	32.59 ± 5.70		
Kheshie 2014	UK	RCT	HILT	20	–	52.1 ± 6.47	29.94 ± 3.36	6 weeks	WOMAC, VAS
			LLLT	18	–	56.56 ± 7.86	28.62 ± 5.2		
			Placebo	15	–	55.6 ± 11.02	28.51 ± 3.35		
Khosravi 2021	Iran	RCT	Knee brace	7	3/4	59.2 ± 8.07	–	6 weeks	WOMAC, VAS
			Lateral wedge insole	7	4/3	60.3 ± 5.28	–		
Khruakhorn 2021	Thailand	RCT	Placebo	17	1/16	57.88 ± 7.75	27.27 ± 4.38	24 weeks	WOMAC
			Hydrotherapy	17	2/15	64.88 ± 7.44	26.34 ± 2.7		
Kilic 2020	Turkey	RCT	Exercise	25	–	59.52 ± 8.57	30.58 ± 2.94	6 weeks	WOMAC, VAS
			Placebo	25	–	60.48 ± 7.43	31.19 ± 3.33		
Kitano 2023	Japan	RCT	Ultrasound	13	3/10	63.5 ± 8.6	26.5 ± 4.3	10 weeks	WOMAC, VAS
			Placebo	13	2/11	56.5 ± 7.5	24.0 ± 5.2		
Kocyigit 2015	Turkey	RCT	KT	21	2/19	52 ± 7.5	–	2 weeks	VAS
			Placebo	20	3/17	52 ± 10	–		
Laufer 2005	Israel	RCT	Short wave diathermy	38	7/31	74.79 + 6.58	–	12 weeks	WOMAC, VAS
			Placebo	33	11/22	73.33 + 6.91	–		
Lee 2023	Korea	RCT	Exercise	15	–	65.63 + 3.70	–	8 weeks	VAS
			Placebo	16	–	68.27 + 4.78	–		
Leon 2017	Mexico	RCT	KT	16	–	56.5 ± 5.0	29.5 ± 4.1	6 weeks	WOMAC, VAS
			Placebo	16	–	59.6 ± 5.2	29.4 ± 3.2		
Lewinson 2016	Canada	RCT	Lateral wedge insole	19	6/13	59.9 ± 7.4	32.5 ± 8.0	12 weeks	VAS
			Placebo	19	8/11	59.6 ± 7.7	29.2 ± 6.7		
Liao 2020	Taiwan	RCT	LLLT	15	1/14	70.53 ± 6.89	26.61 ± 4.33	4 weeks	VAS
			Placebo	15	2/13	69.73 ± 6.91	25.98 ± 2.71		
Lin 2022	Taiwan	RCT	Exercise	20 18	1/19 2/16	75.6 ± 4.4 76.0 ± 5.6	24.6 ± 3.5 24.2 ± 2.2	12 weeks	WOMAC
			Placebo						
Maheu 2022	France	RCT	TENS Placebo	55 55	18/37 21/34	66.9 ± 8.1 66.0 ± 7.8	28.0 ± 5.0 29.8 ± 5.8	12 weeks	WOMAC
Mahler 2018	France	RCT	LLLT Placebo	27 28	12/15 15/13	62 ± 9 68 ± 9	29(25–30) 26(24–31)	12 weeks	VAS
Marconcin 2021	Portugal	RCT	Exercise Placebo	32 35	14/18 7/28	67.8 ± 5.3 70.3 ± 6.1	30.1 ± 5.3 32.3 ± 5.0	–	VAS
Mascarin 2012	Brazil	RCT	Exercise TENS Ultrasound	16 12 12	– – –	59.6 ± 7.2 64.8 ± 7.0 62.8 ± 7.6	– – –	12 weeks	WOMAC, VAS
McManus 2021	Australia	RCT	Placebo KT	17 19	5/12 9/10	70 ± 8.0 68 ± 8.8	30.60 ± 5.0 30.11 ± 5.5	5 weeks	VAS
Messier 2022	USA	RCT	Exercise Placebo	414 409	94/320 92/317	64.5 ± 7.8 64.7 ± 7.8	36.7 ± 6.5 36.9 ± 7.2	72 weeks	WOMAC
Messier 2022	USA	RCT	Exercise Placebo	94 90	30/64 24/66	67.4 ± 6.1 65.0 ± 5.6	33.1 ± 3.3 33.8 ± 3.6	240 weeks	WOMAC
Mete 2022	Turkey	RCT	Exercise Placebo	30 30	6/24 7/23	59.5(55-64) 57(51-65)	30.3 (24.96-33.16) 32.14 (31.8-32.42)	6 weeks	VAS
Mobina 2019	Iran	RCT	Knee brace Lateral edge insole	7 7	3/4 4/3	59.2 ± 8.07 60.3 ± 5.28	– –	6 weeks	WOMAC, VAS
Mohamed 2022	Saudi Arabia	RCT	KT Placebo	20 20	20/0 20/0	60.60 ± 9.43 63.40 ± 7.98	26.65 ± 2.85 27.72 ± 2.41	6 weeks	WOMAC
MohammedSadiq 2021	Iraq	RCT	Exercise Placebo	16 15	3/13 1/14	51.38 ± 7.72 53.8 ± 8.46	31.61 ± 4.12 34.14 ± 4.45	8 weeks	WOMAC
Mostafa 2021	Egypt	RCT	ESWT HILT	20 20	9/11 10/10	40.12 ± 9.45 46.62 ± 8.68	28.82 ± 5.23 29.26 ± 2.48	4 weeks	WOMAC, VAS
Müller-Rath 2011	US	RCT	Knee brace Placebo	13 10	– –	30–60 30–60	< 30 < 30	16 weeks	WOMAC, VAS
Nambi 2016	Saudi Arabia	RCT	LLLT Placebo	17 17	– –	58 ± 6 60 ± 8	26.9 ± 4.8 28.3 ± 3.5	8 weeks	VAS
Nazari 2018	Iran	RCT	HILT Exercise	30 30	13/17 14/16	61.5 ± 3.9 62.24 ± 3.87	27.7 ± 1.4 27.5 ± 1.8	12 weeks	WOMAC
Oğuz 2021	Turkey	RCT	KT Placebo	11 11	0/11 0/22	48.18 ± 7.56 51.00 ± 3.69	30.90 ± 3.17 34.76 ± 5.91	6 weeks	WOMAC, VAS
Palmer 2014	UK	RCT	TENS Placebo	73 74	26/47 25/49	61.2 ± 11.4 60.9 ± 10.8	29.7 ± 11.1 29.1 ± 9.0	24 weeks	WOMAC
Park 2021	South Korea	RCT	Exercise Placebo	25 25	– –	66.88 ± 4.61 68.04 ± 4.16	– –	8 weeks	WOMAC
Pierosimone 2020	US	RCT	TENS Placebo	32 29	14/18 10/19	60.8 ± 7.3 62.5 ± 7.7	29.2 ± 3.3 27.8 ± 4.4	8 weeks	WOMAC
Pinto 2020	Brazil	RCT	LLLT Placebo	15 16	1/14 1/15	63 ± 2.83 66 ± 2.69	24.8 ± 2.4 29.8 ± 1.1	5 weeks	VAS
Pozsgai 2022	Hungary	RCT	Exercise Placebo	21 20	7/14 2/18	68.18 ± 5.21 66.64 ± 4.26	28.62 ± 5.39 32.20 ± 5.73	6 days	WOMAC
Qiestad 2023	Norway	RCT	Exercise Placebo	53 54	25/28 30/24	57.3 ± 7.1 57.8 ± 7.4	29.4 ± 4.4 28.4 ± 4.1	48 weeks	WOMAC
Rabiei 2023	Iran	RCT	Exercise Placebo	27 27	9/18 13/14	60.5 ± 5.6 59.8 ± 5.1	29.5 ± 4.4 29.3 ± 3.4	8 weeks	WOMAC
Rafiq 2021	Malaysia	RCT	Exercise Placebo	28 28	11/17 9/19	54.21 ± 5.20 55.00 ± 4.86	33.91 ± 5.66 31.03 ± 2.78	12 weeks	WOMAC
Rahlf 2017	Germany	RCT	KT Placebo	47 47	23/24 23/24	64.7 ± 7.3 65.3 ± 6.0	– –	3 days	WOMAC
Rego 2023	Brazil	RCT	Exercise Placebo	8 9	0/8 0/9	65.75 ± 2.76 64.78 ± 2.17	28.90 ± 1.41 32.02 ± 2.40	–	VAS
Reichenbach 2021	Switzerland	RCT	TENS Placebo	108 112	56/52 52/60	64.8 ± 9.9 66.3 ± 10.3	27.5 ± 4.9 26.9 ± 4.9	3 weeks	WOMAC
Rewald 2019	Netherlands	RCT	Exercise Placebo	47 55	23/24 16/39	61 ± 7.4 59 ± 9.5	29 ± 5.4 29 ± 5.6	6 weeks	WOMAC
Ridvan 2020	Turkey	RCT	Short wave diathermy Placebo	31 32	14/17 13/19	62.78 ± 7.53 58.68 ± 8.15	– –	12 weeks	WOMAC
Robbins 2021	Australia	RCT	LLLT Placebo	43 43	11/32 5/38	66.09 ± 5.89 62.44 ± 3.34	32.89 ± 5.95 31.69 ± 3.79	11 weeks	WOMAC
Samaan 2022	Egypt	RCT	HILT Ultrasound Placebo	20 20 20	9/11 7/13 10/10	55.4 ± 6.34 55.2 ± 4.77 57 ± 6.39	28.98 ± 2.23 29.1 ± 2.42 29.75 ± 2.12	2 weeks	WOMAC, VAS
Santana ·2022	Austria	RCT	Exercise Placebo	13 13	0/13 0/13	60 ± 10.8 61 ± 6.6	35.3 ± 6.6 33.1 ± 7.3	6 weeks	WOMAC
Sattari 2011	Iran	RCT	Lateral edge insole Knee brace Placebo	20 20 20	– – –	35-65 35-65 35-65	– – –	36 weeks	VAS
Sawitzke 2022	US	RCT	Ultrasound Placebo	67 65	6/61 7/58	62.9 ± 10.5 64.4 ± 10.9	31.8 ± 5.4 31.6 ± 5.5	48 weeks	WOMAC, VAS
Schwartz 2023	Israel	RCT	Lateral edge insole Placebo	26 12	9/17 8/4	67.5 ± 8.8 64.6 ± 8.0	– –	12 weeks	WOMAC, VAS
Sedaghatnezhad 2019	Iran	RCT	Exercise Placebo	15 15	1/14 4/11	53.8 ± 7.43 59.6 ± 7.43	26.55 ± 2.08 27.7 ± 1.93	20 days	VAS
Shah 2022	Saudi Arabia	RCT	KT Placebo	20 20	13/7 14/6	55.55 ± 3.80 55.3 ± 3.88	– –	4 weeks	WOMAC, VAS
Shen 2019	China	RCT	Exercise Placebo	14 13	4/10 4/9	65.3 ± 4.6 66.6 ± 7.0	27.3 ± 2.7 26.5 ± 3.4	6 weeks	VAS
Silva 2007	Brazil	RCT	Hydrotherapy Exercise	32 32	2/30 3/29	59 ± 7.60 59 ± 6.08	– –	18 weeks	WOMAC, VAS
Siriratna 2022	Thailand	RCT	HILT Placebo	21 21	3/18 5/16	66.1 ± 9.4 65.0 ± 8.5	28.1 ± 5.2 27.4 ± 5.8	12 weeks	WOMAC, VAS
Stausholm 2022	Norway	RCT	LLLT Placebo	26 24	8/18 5/19	64.04 ± 8.52 61.92 ± 6.39	28.11 ± 4.31 27.66 ± 3.58	48 weeks	WOMAC, VAS
Tascioglu 2004	Turkey	RCT	LLLT Placebo	20 20	5/15 7/13	59.92 ± 7.59 64.27 ± 10.55	28.63 ± 6.48 29.56 ± 9.54	24 weeks	WOMAC, VAS
Thoumie 2018	France	RCT	knee brace Placebo	32 35	8/24 15/20	64.8 ± 11.7 66.6 ± 7.2	29.2 ± 4.4 28.1 ± 5.1	6 weeks	VAS
Uematsu 2021	Japan	RCT	Ultrasound Placebo	37 33	– –	67-78 68-78	(23.88-28.44) (21.92-26.94)	12 weeks	VAS
Uysal 2020	Turkey	RCT	ESWT Placebo	52 52	10/42 9/43	60.2 ± 6.3 61.8 ± 6.0	30.6 ± 4.3 30.8 ± 4.6	12 weeks	WOMAC, VAS
Vader 2020	Canada	RCT	HILT Placebo	10 10	3/7 4/6	60.60 ± 10.35 67.30 ± 7.01	– –	4 weeks	WOMAC, VAS
Vance 2012	US	RCT	TENS Placebo	25 25	9/16 9/16	55 ± 14.4 57 ± 10.9	36.2 ± 6.0 39.2 ± 7.0	–	VAS
Van 2010	Netherlands	RCT	Lateral wedged insole Knee brace	45 46	20/25 11/35	54.4 ± 6.5 54.9 ± 7.4	29.4 ± 4.9 29.0 ± 4.2	24 weeks	WOMAC, VAS
Vassao 2019	Brazil	RCT	LLLT Exercise	17 15	0/17 0/15	61.65 ± 4.28 65.37 ± 4.19	30.00 ± 3.43 27.52 ± 3.31	16 weeks	VAS
Vassao 2020	Brazil	RCT	LLLT Placebo	17 16	0/17 0/16	61.65 ± 4.42 61.19 ± 4.45	30.00 ± 3.53 30.44 ± 4.76	8 weeks	WOMAC, VAS
Vassao 2021	Brazil	RCT	LLLT Exercise Placebo	13 13 10	0/13 0/13 0/10	62.29 ± 4.39 61.57 ± 4.42 66.5 ± 4.06	30.11 ± 3.64 30.49 ± 4.35 27.24 ± 2.99	8 weeks	WOMAC, VAS
Vincent 2020	US	RCT	Exercise Placebo	19 17	7/12 5/12	66.8 ± 5.4 68.6 ± 7.1	28.7 ± 6.6 32.8 ± 18.2	16 weeks	VAS
Wageck 2016	Australia	RCT	KT Placebo	38 38	3/35 7/31	69.6 ± 6.9 68.66 ± 6.3	30.0 ± 4.9 31.3 ± 4.1	19 days	WOMAC
Ye 2020	China	RCT	Exercise Placebo	28 28	11/17 8/20	65.11 ± 6.57 63.61 ± 2.63	24.19 ± 2.37 24.63 ± 2.27	12 weeks	WOMAC
Yu 2016	Australia	RCT	Knee brace Placebo	50 68	20/30 22/46	67.2 ± 9.6 67.0 ± 10.6	29.6 ± 5.8 33.2 ± 7.4	48 weeks	VAS
Yurtkuran 2007	Turkey	RCT	LLLT Placebo	27 26	1/26 1/25	51.83 ± 6.83 53.478 ± 7.13	31.76 ± 8.81 32.72 ± 3.71	10 weeks	WOMAC, VAS
Zhang 2021	China	RCT	ESWT Placebo	19 14	8/11 6/8	60.84 ± 8.36 61.50 ± 5.43	24.83 ± 1.73 24.98 ± 1.32	4 weeks	WOMAC, VAS

Note: RCT = randomized controlled trial; LLLT = low level laser therapy; HILT = high intensity laser therapy; TENS = transcutaneous electrical nerve stimulation; IFC = interferential current; KT = kinesio taping; ESWT = extracorporeal shock wave therapy.

### 3.2 Risk of bias and quality assessment

The quality assessment of the included 139 RCTs was conducted using the Cochrane Collaboration’s “Risk of Bias” tool. The risk of bias assessment for the included studies is presented in **Table 2**.

**Table 2:** Risk of bias of the included randomized controlled trials.

study	Sequence_generation	Allocation_concealment	Blinding			Selective_reporting-bias	Attrition_bias
			participant	therapist	assessor		
Adıgüzel 2021	computer-generated random number list	unclear	no	no	yes	low risk	low risk
Ahmad 2023	computer-generated randomization table	sealed opaque envelope	yes	no	yes	low risk	low risk
Ahmad 2023	computer-generated randomization table	sealed opaque envelope	no	no	yes	low risk	low risk
Akaltun 2021	envelope pulling procedure	unclear	yes	no	yes	low risk	low risk
Akyol 2010	unclear	unclear	no	no	no	low risk	low risk
Alfredo 2020	permuted block randomization method	unclear	yes	no	yes	low risk	low risk
Alfredo 2022	computer-generated random number table	sealed opaque envelope	yes	yes	yes	low risk	low risk
Alghadir 2013	unclear	sealed opaque envelope	no	no	yes	low risk	low risk
Ali 2014	unclear	unclear	no	no	no	low risk	low risk
Alireza 2023	unclear	sealed opaque envelope	no	no	yes	low risk	low risk
Alkhawajah 2019	randomization procedure.	sealed opaque envelope	yes	no	yes	low risk	low risk
Allen 2021	unclear	unclear	no	no	no	low risk	low risk
Alqualo-Costa 2021	unclear	sealed opaque envelope	yes	no	yes	low risk	low risk
Rashoud 2014	computer-generated randomization table	unclear	yes	no	yes	low risk	low risk
Amornthep 2023	unclear	unclear	yes	no	yes	low risk	low risk
Anandkumar 2014	unclear	sealed opaque envelope	yes	no	yes	low risk	low risk
Anwer 2014	unclear	unclear	no	no	no	low risk	low risk
Artuç 2023	computer-generated randomization table	unclear	yes	no	yes	low risk	low risk
Assar 2020	unclear	unclear	no	no	yes	low risk	low risk
Atamaz 2012	unclear	unclear	yes	yes	yes	low risk	low risk
Aydogdu 2017	unclear	sealed opaque envelope	no	no	yes	low risk	low risk
Baykal 2023	card drawing method	unclear	yes	no	yes	low risk	low risk
Bennell 2015	computer-generated random number table	sealed opaque envelope	yes	no	yes	low risk	low risk
Bennell 2011	random number table	unclear	yes	no	yes	low risk	low risk
Bruce-Brand 2012	unclear	unclear	no	no	yes	low risk	low risk
Cantista 2020	computer-generated randomization table	unclear	no	no	yes	low risk	low risk
Carpenedo 2021	unclear	unclear	yes	no	yes	low risk	low risk
Chen 2014	unclear	sealed opaque envelope	yes	yes	yes	low risk	low risk
Chen 2023	computer-generated random number table	sealed opaque envelope	yes	yes	yes	low risk	low risk
Chen HX 2021	computer-generated randomization table	unclear	no	no	yes	low risk	low risk
Chen PY 2021	unclear	unclear	no	no	yes	low risk	low risk
Cherian 2016	unclear	unclear	yes	no	yes	low risk	low risk
Cho 2015	unclear	sealed opaque envelope	yes	yes	yes	low risk	low risk
Choi 2023	unclear	unclear	no	no	yes	low risk	low risk
Danazumi 2021	block randomization	unclear	yes	no	yes	low risk	low risk
Dantas 2023	unclear	sealed opaque envelope	yes	no	yes	low risk	low risk
Dias 2016	computer randomization	unclear	yes	no	yes	low risk	low risk
Dogan 2022	unclear	sealed opaque envelope	yes	yes	yes	low risk	low risk
Donec 2020	a computer-generated list.	sealed opaque envelope	yes	no	yes	low risk	low risk
Ekici 2022	computer-generated random number table	sealed opaque envelope	yes	no	yes	low risk	low risk
Elboim‑Gabyzon 2023	computer-generated random allocation	unclear	no	no	yes	low risk	low risk
Fazli 2023	permuted block randomization	sealed opaque envelope	yes	no	yes	low risk	low risk
Fokmare 2023	unclear	sealed opaque envelope	yes	no	yes	low risk	low risk
Foley 2014	computer generated randomisation list	sealed opaque envelope	yes	no	yes	low risk	low risk
Foroughi 2011	computerized randomization program	unclear	no	no	yes	low risk	low risk
Fukuda 2011	unclear	sealed opaque envelope	yes	no	yes	low risk	low risk
Fukuda 2011	unclear	sealed opaque envelope	yes	no	yes	low risk	low risk
Fung 2021	unclear	unclear	yes	no	yes	low risk	low risk
Gao 2023	computer-generated.	sealed opaque envelope	no	no	yes	low risk	low risk
Gholami 2023	unclear	unclear	no	no	yes	low risk	low risk
Gomes 2020	unclear	sealed opaque envelope	yes	no	yes	low risk	low risk
Günaydin 2022	unclear	unclear	no	no	no	low risk	low risk
Guo 2021	computer-generated.	unclear	yes	no	yes	low risk	low risk
Gur 2003	unclear	unclear	yes	no	yes	low risk	low risk
Hammam 2020	unclear	sealed opaque envelope	yes	no	yes	low risk	low risk
Han 2021	computer-generated.	sealed opaque envelope	yes	yes	yes	low risk	low risk
Hinman 2003	unclear	unclear	yes	no	yes	low risk	low risk
Ho 2022	computer-generated random number table	unclear	yes	no	yes	low risk	low risk
Hu 2019	computer-generated.	unclear	no	no	yes	low risk	low risk
Iijima 2020	unclear	unclear	no	no	yes	low risk	low risk
Imamura 2016	computer-generated.	sealed opaque envelope	yes	no	yes	low risk	low risk
Itoh 2008	block randomised procedure	unclear	no	no	yes	low risk	low risk
Jang 2023	block randomization technique	sealed opaque envelope	yes	no	yes	low risk	low risk
Jia 2022	unclear	sealed opaque envelope	yes	no	yes	low risk	low risk
Jones 2013	unclear	unclear	no	no	no	low risk	low risk
Jorge 2023	computer-generated.	sealed opaque envelope	yes	no	yes	low risk	low risk
Karakas 2020	block randomization method	sealed opaque envelope	yes	no	yes	low risk	low risk
Karimi 2021	unclear	unclear	no	no	no	low risk	low risk
Kayamutlu 2016	computer-generated.	unclear	yes	no	yes	low risk	low risk
Kayamutlu 2018	computer-generated.	sealed opaque envelope	no	no	yes	low risk	low risk
Kheshie 2014	specific identification number	unclear	yes	no	yes	low risk	low risk
Khosravi 2021	unclear	unclear	no	no	yes	low risk	low risk
Khruakhorn 2021	computer-generated.	sealed opaque envelope	yes	no	yes	low risk	low risk
Kilic 2020	unclear	unclear	no	no	yes	low risk	low risk
Kitano 2023	generated on a computer	sealed opaque envelope	yes	no	yes	low risk	low risk
Kocyigit 2015	the numbered envelopes method	unclear	yes	no	yes	low risk	low risk
Laufer 2005	unclear	sealed opaque envelope	yes	no	yes	low risk	low risk
Lee 2023	random number table	unclear	yes	no	yes	low risk	low risk
Leon 2017	unclear	sealed opaque envelope	yes	no	yes	low risk	low risk
Lewinson 2016	computer-generated.	sealed opaque envelope	yes	no	yes	low risk	low risk
Liao 2020	random number table	sealed opaque envelope	yes	no	yes	low risk	low risk
Lin 2022	unclear	unclear	no	no	yes	low risk	low risk
Maheu 2022	computer-generated.	sealed opaque envelope	yes	no	yes	low risk	low risk
Mahler 2018	stratified block randomisation	sealed opaque envelope	yes	no	yes	low risk	low risk
Marconcin 2021	unclear	sealed opaque envelope	no	no	yes	low risk	low risk
Mascarin 2012	unclear	unclear	yes	no	yes	low risk	low risk
McManus 2021	unclear	unclear	no	no	no	low risk	low risk
Messier 2022	random permuted-block randomization	unclear	no	no	yes	low risk	low risk
Messier 2022	random permuted-block randomization	unclear	no	no	yes	low risk	low risk
Mete 2022	computer-generated	unclear	no	no	yes	low risk	low risk
Mobina 2019	unclear	unclear	yes	no	yes	low risk	low risk
Mohamed 2022	unclear	unclear	yes	no	yes	low risk	low risk
MohammedSadiq 2021	computer-generated	unclear	yes	no	yes	low risk	low risk
Mostafa 2021	unclear	sealed opaque envelope	yes	no	yes	low risk	low risk
Müller-Rath 2011	unclear	unclear	no	no	yes	low risk	low risk
Nambi 2016	unclear	sealed opaque envelope	yes	no	yes	low risk	low risk
Nazari 2018	computer-generated	unclear	yes	yes	yes	low risk	low risk
Oğuz 2021	unclear	sealed opaque envelope	no	no	yes	low risk	low risk
Palmer 2014	unclear	sealed opaque envelope	yes	no	yes	low risk	low risk
Park 2021	random number tables	unclear	no	no	yes	low risk	low risk
Pierosimone 2020	unclear	sealed opaque envelope	yes	no	yes	low risk	low risk
Pinto 2020	computer-generated	unclear	yes	no	yes	low risk	low risk
Pozsgai 2022	unclear	sealed opaque envelope	yes	no	yes	low risk	low risk
Qiestad 2023	Computer-generated randomization lists	unclear	yes	no	yes	low risk	low risk
Rabiei 2023	computer-generated	sealed opaque envelope	no	no	yes	low risk	low risk
Rafiq 2021	computer-generated	unclear	no	no	yes	low risk	low risk
Rahlf 2017	unclear	sealed opaque envelope	yes	no	yes	low risk	low risk
Rego 2023	unclear	sealed opaque envelope	no	no	yes	low risk	low risk
Reichenbach 2021	computer-generated	unclear	no	no	yes	low risk	low risk
Rewald 2019	computer-generated	unclear	no	no	yes	low risk	low risk
Ridvan 2020	unclear	unclear	yes	no	yes	low risk	low risk
Robbins 2021	computer-generated	unclear	yes	no	yes	low risk	low risk
Samaan 2022	computer-generated randomized table	unclear	no	no	yes	low risk	low risk
Santana 2022	unclear	unclear	no	no	yes	low risk	low risk
Sattari 2011	computer-generated procedure	unclear	no	no	yes	low risk	low risk
Sawitzke 2022	random block sizes	unclear	yes	no	yes	low risk	low risk
Schwartz 2023	unclear	unclear	yes	no	yes	low risk	low risk
Sedaghatnezhad 2019	flipping a coin	unclear	yes	no	yes	low risk	low risk
Shah 2022	computer-generated	sealed opaque envelope	yes	no	yes	low risk	low risk
Shen 2019	computer-generated	sealed opaque envelope	no	no	yes	low risk	low risk
Silva 2007	drawing lots	unclear	no	no	yes	low risk	low risk
Siriratna 2022	unclear	sealed opaque envelope	no	no	yes	low risk	low risk
Stausholm 2022	unclear	sealed opaque envelope	yes	no	yes	low risk	low risk
Tascioglu 2004	numbered envelopes	unclear	yes	no	yes	low risk	low risk
Thoumie 2018	unclear	unclear	no	no	yes	low risk	low risk
Uematsu 2021	unclear	sealed opaque envelope	yes	yes	yes	low risk	low risk
Uysal 2020	unclear	unclear	no	no	yes	low risk	low risk
Vader 2020	computer-generated	sealed opaque envelope	yes	no	yes	low risk	low risk
Vance 2012	unclear	sealed opaque envelope	yes	no	yes	low risk	low risk
Van 2010	Computer-generated procedure	unclear	no	no	yes	low risk	low risk
Vassao 2019	a random table of numbers	unclear	yes	no	yes	low risk	low risk
Vassao 2020	computer-generated	sealed opaque envelope	yes	no	yes	low risk	low risk
Vassao 2021	random table of numbers	unclear	yes	no	yes	low risk	low risk
Vincent 2020	computer-generated	sealed opaque envelope	no	no	yes	low risk	low risk
Wageck 2016	unclear	sealed opaque envelope	no	no	yes	low risk	low risk
Ye 2020	computer-generated	sealed opaque envelope	no	no	yes	low risk	low risk
Yu 2016	unclear	unclear	no	no	yes	low risk	low risk
Yurtkuran 2007	computer-generated	unclear	yes	no	yes	low risk	low risk
Zhang 2021	computer-generated	sealed opaque envelope	yes	no	yes	low risk	low risk

### 3.3 Evidence network

**Fig 2:**
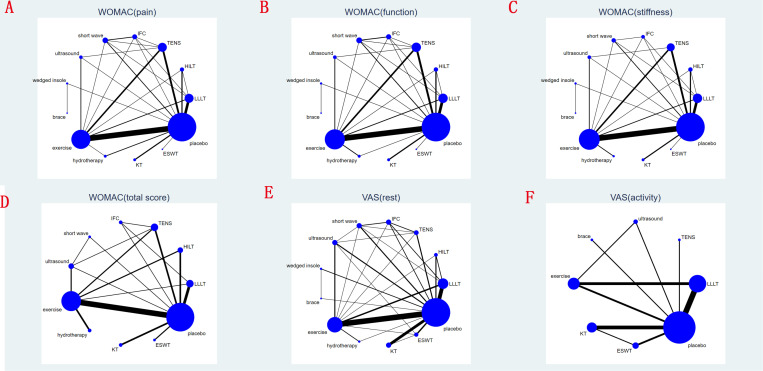
Network analysis of eligible comparison for (A) WOMAC pain score, (B) WOMAC function score, (C) WOMAC stiffness score, (D) total WOMAC score, (E) VAS-rest and (F) VAS-activity at last follow-up. The size of each node represents the number of participants, while the thickness of the line represents the number of studies directly comparing the two interventions.

This study encompassed 12 distinct physical therapies, including LLLT, HILT, TENS, IFC, short wave diathermy, ultrasound, lateral wedged insole, knee brace, exercise, hydrotherapy, KT and ESWT. **Fig 2** visually represents the evidence network, where the lines denote direct comparisons between two directly related interventions. Interventions lacking direct connections are compared indirectly through the network meta-analysis. The width of the lines reflects the number of trials, while the size of the nodes corresponds to the total sample size across multiple treatments.

### 3.4 Inconsistency test

**Fig 3:**
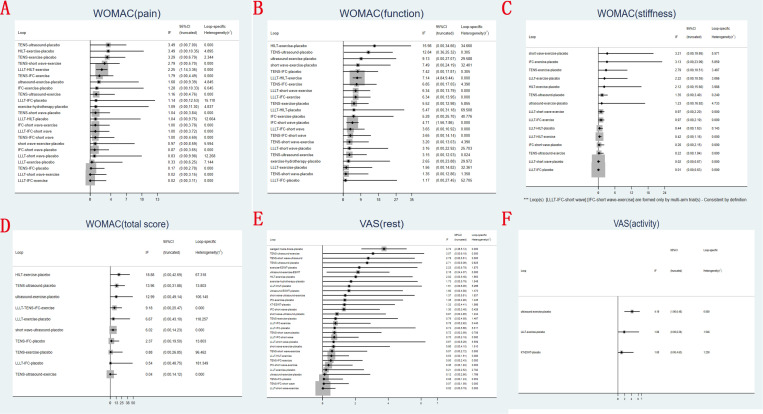
Inconsistency plot of eligible comparison for (A) WOMAC pain score, (B) WOMAC function score, (C) WOMAC stiffness score, (D) total WOMAC score, (E) VAS-rest and (F) VAS-activity at last follow-up.

**Fig 3** displays an inconsistency plot designed to assess heterogeneity among studies within the closed loops of the network meta-analysis. For WOMAC pain score, there were 23 closed loops, with IF ranging from 0.02 to 3.49. The majority of these closed loops had 95%CIs that contained 0, and only one closed loops of LLLT~HILT~exercise had 95%CIs approaching 0. Similarly, regarding WOMAC function score, there were 22 closed loops, with IF ranging from 1.17 to 15.98, and the majority of these closed loops had 95%CIs that contained 0. Only three closed loops had 95%CIs approaching 0, including loops of TENS~ultrasound~placebo, LLLT~HILT~exercise, and IFC~short wave diathermy ~ placebo. In terms of the WOMAC stiffness score, there were 15 closed loops, with IF ranging from 0.01 to 3.21, all of which were close to 0. Likewise, for the total WOMAC score, there were 10 closed loops, with IF ranging from 0.04 to 18.88, all of which were close to 0. The 95%CIs for these IF values contained 0, indicating no statistically significant differences. For VAS-rest, there were 31 closed loops, with IF ranging from 0.02 to 3.74, and the majority of these closed loops had 95%CIs that contained 0. Only two closed loops had 95%CIs approaching 0, including loops of lateral wedged insole ~ knee brace~placebo, and ultrasound~exercise~ESWT. And in terms of VAS-activity, there were only 3 closed loops, with IF ranging from 1.08 to 4.19. The majority of these closed loops had 95%CIs that contained 0, and only one closed loops of ultrasound~exercise~placebo had 95%CIs approaching 0. Overall, these results suggest that the data exhibited consistency.

### 3.5 Results of Network meta-analysis

#### 3.5.1 WOMAC pain score at last follow-up.

**Fig 4:**
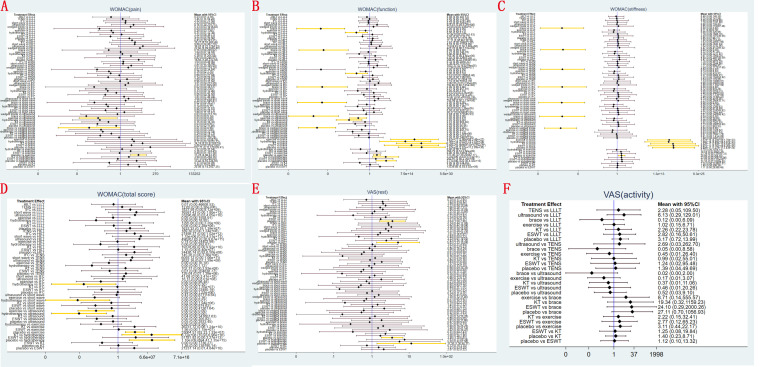
Forest plots for (A) WOMAC pain score, (B) WOMAC function score, (C) WOMAC stiffness score, (D) total WOMAC score, (E) VAS-rest and (F) VAS-activity at last follow-up.

The results of the network meta-analysis revealed the following findings regarding WOMAC pain score at last follow-up: knee brace demonstrated a lower WOMAC pain score compared to lateral wedged insole. Exercise exhibited a lower WOMAC pain score than ultrasound and placebo. Other comparisons did not yield statistically significant differences (**Fig 4** and **Table 3**).

**Table 3:** Comparative primary outcomes for WOMAC pain score, WOMAC function score, WOMAC stiffness score, total WOMAC score, VAS-rest and VAS-activity. Significant results are in bold text.

	LLLT	HILT	TENS	IFC	short wave diathermy	ultrasound	lateral wedged insole	knee brace	exercise	hydrotherapy	KT	ESWT	placebo
**WOMAC pain score**	**LLLT**	0.23 (0.01,4.19)	1.45 (0.11,19.28)	2.32 (0.07,77.56)	2.23 (0.09,52.93)	8.16 (0.24,279.71)	5.13 (0.03,1019.82)	0.04 (0.00,49.97)	0.24 (0.03,1.96)	0.31 (0.01,12.38)	1.22 (0.04,38.77)	0.21 (0.00,42.63)	4.20 (0.65,26.96)
**WOMAC function score**	13.15 (0.01,32425.79)	**HILT**	6.43 (0.29,143.51)	10.27 (0.18,585.45)	9.84 (0.24,407.79)	36.03 (0.72,1794.23)	22.66 (0.09,5905.40)	0.16 (0.00,269.62)	1.04 (0.07,14.93)	1.38 (0.02,79.37)	5.39 (0.11,255.83)	0.92 (0.00,246.57)	18.56 (1.48,232.58)
	1.18 (0.00,1086.82)	0.09 (0.00,373.76)	**TENS**	1.60 (0.05,49.40)	1.53 (0.07,34.48)	5.61 (0.20,160.00)	3.53 (0.02,714.70)	0.02 (0.00,34.84)	0.16 (0.02,1.20)	0.22 (0.01,8.59)	0.84 (0.03,27.44)	0.14 (0.00,29.87)	2.89 (0.43,19.56)
	3.63 (0.00,36657.10)	0.28 (0.00,12702.52)	3.07 (0.00,26899.59)	**IFC**	0.96 (0.03,31.56)	3.51 (0.04,273.59)	2.21 (0.01,825.06)	0.02 (0.00,34.51)	0.10 (0.00,2.80)	0.13 (0.00,12.46)	0.52 (0.01,41.25)	0.09 (0.00,34.40)	1.81 (0.07,46.44)
	3.15 (0.00,10851.37)	0.24 (0.00,4113.66)	2.66 (0.00,8143.64)	0.87 (0.00,7786.47)	**short wave diathermy**	3.66 (0.06,214.81)	2.30 (0.01,688.41)	0.02 (0.00,30.37)	0.11 (0.01,2.00)	0.14 (0.00,9.71)	0.55 (0.01,31.61)	0.09 (0.00,28.73)	1.88 (0.11,31.57)
	0.00 (0.00,12.22)	0.00 (0.00,2.77)	0.00 (0.00,6.22)	0.00 (0.00,29.12)	0.00 (0.00,13.62)	**ultrasound**	0.63 (0.00,213.74)	0.00 (0.00,9.14)	**0.03 (0.00,0.60)**	0.04 (0.00,3.09)	0.15 (0.00,10.32)	0.03 (0.00,8.91)	0.52 (0.02,11.08)
	0.03 (0.00,37523.63)	0.00 (0.00,6311.61)	0.03 (0.00,33320.21)	0.01 (0.00,53421.83)	0.01 (0.00,30727.87)	27.32 (0.00,1.31e + 08)	**lateral wedged insole**	**0.01 (0.00,0.98)**	0.05 (0.00,7.68)	0.06 (0.00,22.14)	0.24 (0.00,74.72)	0.04 (0.00,45.91)	0.82 (0.01,116.20)
	**4.68e + 19 (9.66e + 09,2.27e + 29)**	**3.56e + 18 (4.43e + 08,2.86e + 28)**	**3.96e + 19 (7.91e + 09,1.98e + 29)**	**1.29e + 19 (9.16e + 08,1.81e + 29)**	**1.49e + 19 (1.67e + 09,1.32e + 29)**	**4.10e + 22 (3.47e + 12,4.85e + 32)**	**1.50e + 21 (4.33e + 13,5.20e + 28)**	**knee brace**	6.62 (0.01,8158.15)	8.77 (0.00,19270.33)	34.19 (0.02,67309.15)	5.86 (0.00,31621.69)	117.71 (0.11,129326.41)
	48.69 (0.19,12590.73)	3.70 (0.00,4741.49)	41.14 (0.21,7899.52)	13.41 (0.00,82345.77)	15.47 (0.01,28408.83)	42676.09 (15.25,1.19e + 08)	1561.88 (0.00,1.18e + 09)	**9.61e + 17 (2.66e + 08,3.48e + 27)**	**exercise**	1.32 (0.05,37.54)	5.16 (0.21,124.43)	0.89 (0.01,151.38)	**17.78 (4.98,63.45)**
	**236290.22 (6.94,8.04e + 09)**	17971.55 (0.20,1.58e + 09)	**199673.19 (6.41,6.22e + 09)**	65088.72 (0.25,1.66e + 10)	75055.97 (0.67,8.36e + 09)	**2.07e + 08 (1294.96,3.31e + 13)**	7.58e + 06 (0.76,7.55e + 13)	**1.98e + 14 (10250.79,3.83e + 24)**	4853.19 (0.52,4.55e + 07)	**hydrotherapy**	3.90 (0.05,294.16)	0.67 (0.00,248.00)	13.42 (0.55,326.50)
	0.09 (0.00,820.79)	0.01 (0.00,201.68)	0.07 (0.00,747.27)	0.02 (0.00,2387.19)	0.03 (0.00,1048.55)	76.65 (0.00,5.32e + 06)	2.81 (0.00,1.13e + 07)	**5.35e + 20 (5.07e + 10,5.66e + 30)**	0.00 (0.00,8.06)	0.00 (0.00,0.07)	**KT**	0.17 (0.00,55.09)	3.44 (0.19,63.60)
	835.40 (0.00,7.79e + 08)	63.54 (0.00,1.33e + 08)	705.94 (0.00,6.92e + 08)	230.12 (0.00,1.14e + 09)	265.36 (0.00,6.48e + 08)	732250.27 (0.19,2.78e + 12)	26799.27 (0.00,2.50e + 12)	**5.60e + 16 (600611.67,5.23e + 27)**	17.16 (0.00,9.91e + 06)	0.00 (0.00,28194.29)	9553.50 (0.00,3.05e + 10)	**ESWT**	20.08 (0.14,2927.25)
	0.06 (0.00,8.54)	0.00 (0.00,4.52)	0.05 (0.00,8.27)	0.02 (0.00,87.72)	0.02 (0.00,26.12)	54.89 (0.02,171585.85)	2.01 (0.00,993046.38)	**7.47e + 20 (2.67e + 11,2.09e + 30)**	0.00 (0.00,0.04)	3.77e + 06 (322.45,4.42e + 10)	0.72 (0.00,1604.72)	0.00 (0.00,28.19)	**placebo**
	**LLLT**	**HILT**	**TENS**	**IFC**	**short wave diathermy**	**ultrasound**	**lateral wedged insole**	**knee brace**	**exercise**	**hydrotherapy**	**KT**	**ESWT**	**placebo**
**WOMAC stiffness score**	**LLLT**	0.48 (0.00,148.30)	0.75 (0.00,141.56)	0.89 (0.00,4429.98)	1.19 (0.00,1331.90)	0.88 (0.00,739.83)	2.65 (0.00,119297.48)	**2.49e + 17 (3.16e + 10,1.96e + 24)**	0.11 (0.00,6.70)	0.08 (0.00,326.95)	1.78 (0.00,1685.16)	2.40 (0.00,107240.06)	2.65 (0.07,98.11)
**Total WOMAC score**	14.96 (0.00,1.04e + 07)	**HILT**	1.55 (0.00,753.23)	1.83 (0.00,24067.98)	2.45 (0.00,7641.54)	1.82 (0.00,3232.06)	5.47 (0.00,411249.42)	**1.21e + 17 (1.08e + 10,1.35e + 24)**	0.22 (0.00,41.26)	0.16 (0.00,1217.97)	3.67 (0.00,7560.20)	4.95 (0.00,369705.69)	5.47 (0.04,755.45)
	0.08 (0.00,20352.80)	0.01 (0.00,3453.01)	**TENS**	1.18 (0.00,9875.29)	1.58 (0.00,2844.96)	1.17 (0.00,668.97)	3.54 (0.00,177537.68)	**1.86e + 17 (2.20e + 10,1.58e + 24)**	0.14 (0.00,8.24)	0.10 (0.00,419.57)	2.37 (0.00,2662.01)	3.20 (0.00,159555.92)	3.54 (0.07,178.55)
	0.00 (0.00,2405.14)	0.00 (0.00,1353.35)	0.01 (0.00,15916.11)	**IFC**	1.34 (0.00,14898.07)	0.99 (0.00,20839.05)	2.99 (0.00,1.36e + 06)	**2.21e + 17 (5.41e + 09,9.01e + 24)**	0.12 (0.00,516.25)	0.08 (0.00,5550.31)	2.00 (0.00,48659.97)	2.70 (0.00,1.23e + 06)	2.99 (0.00,11408.22)
	0.00 (0.00,307.41)	0.00 (0.00,34.35)	0.00 (0.00,2892.01)	0.02 (0.00,1.39e + 07)	**short wave diathermy**	0.74 (0.00,4016.05)	2.23 (0.00,360432.11)	**2.95e + 17 (1.54e + 10,5.65e + 24)**	0.09 (0.00,75.96)	0.06 (0.00,1290.42)	1.50 (0.00,9136.64)	2.02 (0.00,324165.82)	2.23 (0.00,1460.44)
	0.00 (0.00,36.51)	0.00 (0.00,3.90)	0.00 (0.00,186.89)	0.06 (0.00,2.44e + 06)	2.30 (0.00,4.61e + 06)	**ultrasound**	3.01 (0.00,340598.42)	**2.19e + 17 (1.47e + 10,3.26e + 24)**	0.12 (0.00,36.85)	0.09 (0.00,896.66)	2.02 (0.00,7502.03)	2.73 (0.00,306253.90)	3.01 (0.01,993.53)
							**lateral wedged insole**	**6.59e + 17 (5.36e + 12,8.11e + 22)**	0.04 (0.00,1326.62)	0.03 (0.00,9267.53)	0.67 (0.00,76921.76)	0.91 (0.00,1.41e + 06)	1.00 (0.00,24074.91)
								**knee brace**	**2.67e + 16 (4.20e + 09,1.70e + 23)**	**1.88e + 16 (5.89e + 08,5.97e + 23)**	**4.42e + 17 (2.94e + 10,6.64e + 24)**	**5.97e + 17 (5.75e + 09,6.20e + 25)**	**6.60e + 17 (1.27e + 11,3.43e + 24)**
	20.26 (0.00,639640.79)	1.35 (0.00,44713.44)	256.24 (0.01,5.04e + 06)	37222.39 (0.01,1.36e + 11)	1.55e + 06 (0.42,5.67e + 12)	**674466.06 (10.58,4.30e + 10)**			**exercise**	0.70 (0.00,1024.14)	16.54 (0.03,9425.20)	22.34 (0.00,725935.33)	**24.69 (2.00,304.16)**
	673702.35 (0.05,8.60e + 12)	45020.33 (0.00,5.93e + 11)	8.52e + 06 (0.89,8.15e + 13)	**1.24e + 09 (3.36,4.55e + 17)**	**5.16e + 10 (141.26,1.89e + 19)**	**2.24e + 10 (1125.11,4.47e + 17)**			33259.16 (0.11,1.05e + 10)	**hydrotherapy**	23.58 (0.00,369768.96)	31.83 (0.00,1.03e + 07)	35.18 (0.02,78094.60)
	0.00 (0.00,1908.50)	0.00 (0.00,264.20)	0.01 (0.00,27972.20)	1.42 (0.00,1.48e + 08)	59.15 (0.00,6.77e + 09)	25.71 (0.00,2.26e + 08)			0.00 (0.00,18.01)	0.00 (0.00,0.09)	**KT**	1.35 (0.00,153897.51)	1.49 (0.00,506.39)
	13.01 (0.00,3.87e + 08)	0.87 (0.00,4.84e + 07)	164.58 (0.00,5.55e + 09)	23907.68 (0.00,1.91e + 13)	996561.45 (0.00,8.69e + 14)	433204.75 (0.00,3.86e + 13)			0.64 (0.00,4.75e + 06)	0.00 (0.00,12192.51)	16848.45 (0.00,2.20e + 12)	**ESWT**	1.11 (0.00,26398.49)
	0.00 (0.00,5.23)	0.00 (0.00,1.09)	0.01 (0.00,83.22)	1.13 (0.00,1.93e + 06)	46.93 (0.00,9.09e + 07)	20.40 (0.00,1.22e + 06)			0.00 (0.00,0.01)	0.00 (0.00,0.00)	0.79 (0.00,87984.00)	0.00 (0.00,107.40)	**placebo**
	**LLLT**	**HILT**	**TENS**	**IFC**	**short wave diathermy**	**ultrasound**	**lateral wedged insole**	**knee brace**	**exercise**	**hydrotherapy**	**KT**	**ESWT**	**placebo**
**VAS-rest**	**LLLT**	0.70 (0.17,2.85)	1.93 (0.44,8.42)	1.99 (0.44,9.05)	2.33 (0.54,10.04)	1.66 (0.38,7.31)	1.95 (0.25,15.09)	1.17 (0.10,13.60)	1.02 (0.40,2.61)	0.31 (0.03,2.92)	2.34 (0.63,8.76)	1.71 (0.35,8.37)	**3.54 (1.58,7.96)**
**VAS-activity**		**HILT**	2.74 (0.44,17.08)	2.82 (0.42,18.94)	3.30 (0.53,20.65)	2.35 (0.38,14.60)	2.77 (0.28,27.70)	1.67 (0.12,24.01)	1.44 (0.36,5.87)	0.44 (0.04,5.22)	3.33 (0.61,18.03)	2.43 (0.36,16.35)	**5.02 (1.33,19.04)**
	0.44 (0.01,21.04)		**TENS**	1.03 (0.21,4.99)	1.21 (0.23,6.23)	0.86 (0.16,4.74)	1.01 (0.10,9.87)	0.61 (0.04,8.58)	0.53 (0.13,2.11)	0.16 (0.01,1.88)	1.21 (0.23,6.36)	0.89 (0.14,5.76)	1.83 (0.51,6.66)
				**IFC**	1.17 (0.22,6.19)	0.83 (0.13,5.20)	0.98 (0.09,10.33)	0.59 (0.04,8.89)	0.51 (0.11,2.29)	0.16 (0.01,1.95)	1.18 (0.20,6.84)	0.86 (0.12,6.13)	1.78 (0.43,7.36)
					**short wave diathermy**	0.71 (0.14,3.59)	0.84 (0.09,8.25)	0.50 (0.04,7.16)	0.44 (0.11,1.77)	0.13 (0.01,1.57)	1.01 (0.19,5.32)	0.74 (0.11,4.77)	1.52 (0.41,5.60)
	0.16 (0.01,3.44)		0.37 (0.00,36.41)			**ultrasound**	1.18 (0.12,11.45)	0.71 (0.05,9.97)	0.61 (0.16,2.35)	0.19 (0.02,2.15)	1.41 (0.27,7.32)	1.03 (0.17,6.11)	2.13 (0.59,7.72)
							**lateral wedged insole**	0.60 (0.06,5.92)	0.52 (0.07,3.83)	0.16 (0.01,2.72)	1.20 (0.14,10.28)	0.88 (0.09,9.00)	1.81 (0.28,11.86)
	8.54 (0.16,444.61)		19.49 (0.12,3258.42)			52.35 (0.50,5483.52)		**knee brace**	0.87 (0.08,9.64)	0.27 (0.01,6.13)	2.00 (0.16,25.23)	1.46 (0.10,21.53)	3.02 (0.30,30.47)
	0.98 (0.15,6.46)		2.24 (0.04,132.28)			6.01 (0.33,111.14)		0.11 (0.00,7.33)	**exercise**	0.31 (0.04,2.43)	2.30 (0.67,7.92)	1.68 (0.38,7.46)	**3.48 (1.78,6.81)**
										**hydrotherapy**	7.53 (0.71,80.24)	5.50 (0.45,67.98)	**11.38 (1.36,95.37)**
	0.44 (0.04,4.64)		1.01 (0.02,55.86)			2.71 (0.09,81.03)		0.05 (0.00,3.10)	0.45 (0.03,6.57)		**KT**	0.73 (0.14,3.75)	1.51 (0.53,4.28)
	0.35 (0.02,6.36)		0.81 (0.01,62.44)			2.17 (0.05,95.62)		0.04 (0.00,3.44)	0.36 (0.02,8.51)		0.80 (0.05,12.78)	**ESWT**	2.07 (0.52,8.22)
	0.32 (0.07,1.39)		0.72 (0.02,25.68)			1.93 (0.11,33.94)		0.04 (0.00,1.44)	0.32 (0.05,2.29)		0.71 (0.11,4.43)	0.89 (0.08,10.52)	**placebo**

**Fig 5:**
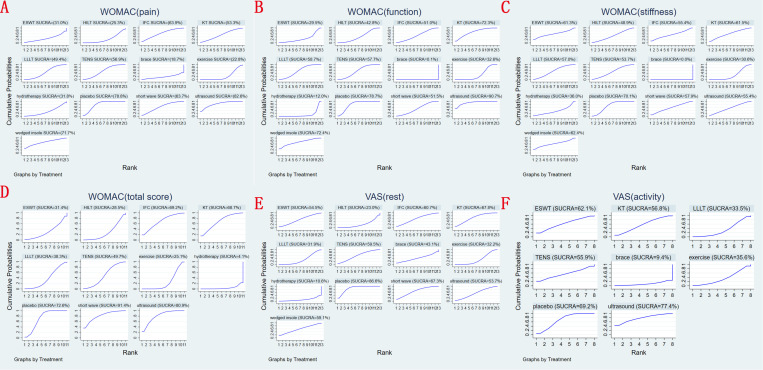
Surface under the cumulative ranking (SUCRA) for (A) WOMAC pain score, (B) WOMAC function score, (C) WOMAC stiffness score, (D) total WOMAC score, (E) VAS-rest and (F) VAS-activity at last follow-up.

A ranking graph depicting the distribution of probabilities for WOMAC pain score at last follow-up is presented in **Fig 5**. Based on the Surface Under the Cumulative Ranking Curve (SUCRA), knee brace obtained the lowest SUCRA rank, indicating the highest probability of relieving knee pain. Conversely, ultrasound had the lowest probability of relieving knee pain. The SUCRA rankings for WOMAC pain score at last follow-up were as follows: knee brace (18.7%) <exercise (22.8%) <HILT (25.3%) <ESWT (31.0%) <hydrotherapy (31.8%) <LLLT (49.4%) <KT (53.3%) <TENS (56.9%) <short wave diathermy (63.7%) <IFC (63.9%) <lateral wedged insole (71.7%) <placebo (78.8%) <ultrasound (82.6%).

#### 3.5.2 WOMAC function score at last follow-up.

The results of the network meta-analysis indicated the following findings concerning WOMAC function score at last follow-up: knee brace demonstrated a lower WOMAC function score compared to LLLT, HILT, TENS, IFC, short wave diathermy, ultrasound, lateral wedged insole, exercise, hydrotherapy, KT, ESWT and placebo. Hydrotherapy exhibited lower WOMAC function score compared to LLLT, TENS and ultrasound. Exercise showed lower WOMAC function score compared to ultrasound. Other comparisons did not yield statistically significant differences (**Fig 4** and **Table 3**).

A ranking graph depicting the distribution of probabilities for WOMAC function score at last follow-up is presented in **Fig 5**. Based on the SUCRA, knee brace obtained the lowest SUCRA rank, indicating the highest probability of recovering knee function. Conversely, ultrasound had the lowest probability of recovering knee function. The SUCRA rankings for WOMAC function score at last follow-up were as follows: knee brace (0.1%) <hydrotherapy (12%) <ESWT (29.5%) <exercise (32.6%) <HILT (42.8%) <IFC (51%) <short wave diathermy (51.5%) <TENS (57.7%) <LLLT (58.7%) <KT (72.3%) <lateral wedged insole (72.4%) <placebo (78.7%) <ultrasound (90.7%).

#### 3.5.3 WOMAC stiffness score at last follow-up.

The results of the network meta-analysis indicated the following findings concerning WOMAC stiffness score at last follow-up: knee brace demonstrated a lower WOMAC stiffness score compared to LLLT, HILT, TENS, IFC, short wave diathermy, ultrasound, lateral wedged insole, exercise, hydrotherapy, KT, ESWT and placebo. Exercise showed lower WOMAC stiffness score compared to placebo. Other comparisons did not yield statistically significant differences (**Fig 4** and **Table 3**).

A ranking graph depicting the distribution of probabilities for WOMAC stiffness score at last follow-up is presented in **Fig 5**. Based on the SUCRA, knee brace obtained the lowest SUCRA rank, indicating the highest probability of relieving knee stiffness. Conversely, placebo had the lowest probability of relieving knee stiffness. The SUCRA rankings for WOMAC stiffness score at last follow-up were as follows: knee brace (0%) <exercise (30.6%) <hydrotherapy (36%) <HILT (48.9%) <TENS (53.7%) <IFC (55.4%) <ultrasound (55.4%) <LLLT (57%) <short wave diathermy (57.9%) <ESWT (61.3%) <KT (61.5%) <lateral wedged insole (62.4%) <placebo (70.1%).

#### 3.5.4 Total WOMAC score at last follow-up.

The results of the network meta-analysis indicated the following findings concerning total WOMAC score at last follow-up: hydrotherapy demonstrated a lower total WOMAC score compared to IFC, short wave diathermy, ultrasound, KT and placebo. Exercise showed lower total WOMAC score compared to ultrasound and placebo. Other comparisons did not yield statistically significant differences (**Fig 4** and **Table 3**).

A ranking graph depicting the distribution of probabilities for total WOMAC score at last follow-up is presented in **Fig 5**. Based on the SUCRA, hydrotherapy obtained the lowest SUCRA rank, indicating the highest probability. Conversely, short wave diathermy had the lowest probability. The SUCRA rankings for total WOMAC score at last follow-up were as follows: hydrotherapy (4.1%) <exercise (25.1%) <HILT (28.5%) <ESWT (31.4%) <LLLT (38.3%) <TENS (49.7%) <KT (68.7%) <IFC (69.2%) <placebo (72.6%) <ultrasound (80.9%) <short wave diathermy (91.4%).

#### 3.5.5 VAS-rest at last follow-up.

The results of the network meta-analysis indicated the following findings concerning VAS-rest at last follow-up: Placebo demonstrated a higher VAS-rest compared to hydrotherapy, HILT, LLLT, and exercise. Other comparisons did not yield statistically significant differences (**Fig 4** and **Table 3**).

A ranking graph depicting the distribution of probabilities for VAS-rest at last follow-up is presented in **Fig 5**. Based on the SUCRA, hydrotherapy obtained the lowest SUCRA rank, indicating the highest probability. Conversely, placebo had the lowest probability. The SUCRA rankings for VAS-rest at last follow-up were as follows: hydrotherapy (10.6%) <HILT (23%) <LLLT (31.9%) <exercise (32.2%) <knee brace (43.1%) <ultrasound (53.7%) <ESWT (54.5%) <lateral wedged insole (59.1%) <TENS (59.5%) <IFC (60.7%) <short wave diathermy (67.3%) <KT (67.8%) <placebo (86.6%).

#### 3.5.6 VAS-activity at last follow-up.

The results of the network meta-analysis indicated that all comparisons did not yield statistically significant differences concerning VAS-activity at last follow-up (**Fig 4** and **Table 3**).

A ranking graph depicting the distribution of probabilities for VAS-activity at last follow-up is presented in **Fig 5**. Based on the SUCRA, knee brace obtained the lowest SUCRA rank, indicating the highest probability. Conversely, ultrasound had the lowest probability. The SUCRA rankings for VAS-activity at last follow-up were as follows: knee brace (9.4%) <LLLT (33.5%) <exercise (35.6%) <TENS (55.9%) <KT (56.8%) <ESWT (62.1%) <placebo (69.2%) <ultrasound (77.4%).

### 3.6 Publication bias

**Fig 6:**
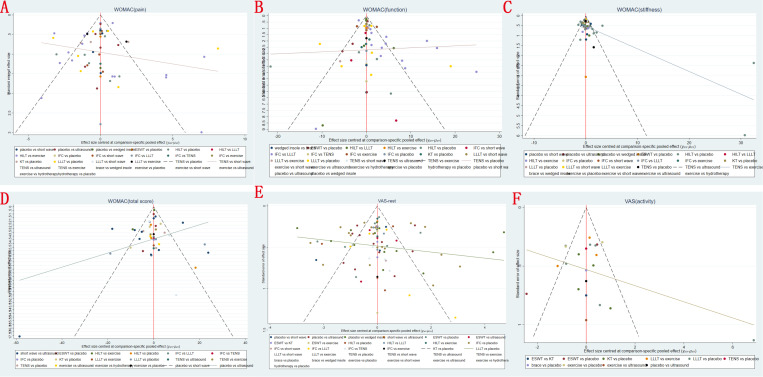
Funnel plots of (A) WOMAC pain score, (B) WOMAC function score, (C) WOMAC stiffness score, (D) total WOMAC score, (E) VAS-rest and (F) VAS-activity at last follow-up.

Based on the outcomes observed for WOMAC pain score, WOMAC function score, WOMAC stiffness score, total WOMAC score, VAS-rest and VAS-activity at last follow-up, network meta-analysis showed that the corrected funnel plots were generated to assess publication bias and potential small sample effects. The analysis revealed that most data points were well-distributed within the funnel plot, displaying relative symmetry on both sides. Additionally, the regression line closely paralleled the X-axis, indicating minimal likelihood of publication bias or small sample effects (**Fig 6**).

## 4 Discussion

Despite evidence from numerous clinical randomized controlled trials and meta-analyses that physical therapy has promising effects on knee osteoarthritis [7–17], a network meta-analysis comprehensively analyzing the clinical efficacy of 12 therapeutic options, including low level laser therapy, high intensity laser therapy, transcutaneous electrical nerve stimulation, interferential current, short wave diathermy, ultrasound, lateral wedged insole, knee brace, exercise, hydrotherapy, kinesio taping and extracorporeal shock wave therapy, is currently lacking. This implies that while we recognize the positive role of physical therapy in improving symptoms and functionality in knee osteoarthritis patients, these methods may differ in mechanisms of action, applicability, and efficacy variances. For instance, LLLT may function by promoting cellular metabolism and tissue repair, whereas HLLT may focus more on alleviating inflammation and pain in deep tissues. TENS works by modulating neural conduction to reduce pain perception. However, due to the absence of more in-depth and comprehensive network meta-analysis, it remains unclear which physical therapy method is most effective for different patient populations and how to optimize the combination of these interventions for the best therapeutic outcomes. This study aims to ascertain the comparative effects of various physical therapies for knee osteoarthritis, aiding clinicians in precisely selecting the most suitable physical therapy method based on individual patient conditions, enhancing treatment efficacy, reducing unnecessary medical resource wastage, and providing robust guidance and evidence for future research directions. Based on 139 included randomized controlled trials, we conducted a focused analysis and discussion of WOMAC and VAS scores. Overall results indicate that knee brace are the most effective, followed by hydrotherapy, exercise, HILT, ESWT, LLLT, TENS, IFC, KT, short wave diathermy, ultrasound, and lateral wedged insole. When considering pain relief in particular, the hierarchy is hydrotherapy, HILT, LLLT, exercise, knee brace, ultrasound, ESWT, lateral wedged insole, TENS, IFC, short wave diathermy, and KT.

On the whole, Knee orthoses provide the most effective treatment for knee osteoarthritis, with their primary mechanisms of action being as follows: (1) Improving joint biomechanics by adjusting the knee joint’s force line to evenly distribute load and reduce excessive stress on cartilage and soft tissues. Knee orthoses can apply a valgus force through a brace or modify the ground reaction force to change the medial knee load [18]. They rapidly enhance the knee’s walking patterns and biomechanical gait efficiency [19–21]. Cudejko [22] and colleagues propose that knee orthoses widen the medial joint space during walking, addressing a primary cause of pain symptoms. (2) Enhancing joint stability by limiting excessive knee movement in unstable conditions, thereby reducing injury risk and pain [23,24]. (3) Alleviating muscle fatigue by supporting surrounding muscles and reducing their workload to maintain joint function. (4) Adjusting proprioception to improve patients’ awareness of knee joint position and movement, enhancing joint control [25]. (5) Reducing the inflammatory response by limiting inflammation spread and easing related symptoms.

For the reduction of pain, this study demonstrates that aquatic therapy is particularly effective. This therapy, which can involve exercise in water or the use of water’s properties for treatment, has been shown by evidence from past studies to significantly reduce pain and enhance physical function through heat stimulation and buoyancy. Water temperatures ranging from 33.5°C to 35.5°C are optimal for allowing prolonged immersion without thermal discomfort and for providing an adequate exercise duration for therapeutic benefits [26–28]. Increased water depth provides greater buoyancy, unloading the joints and consequently easing the pain associated with knee osteoarthritis [29]. Additionally, aquatic therapy addresses multiple psychosocial factors, including depression, self-efficacy, and exercise avoidance [30]. According to Lim’s 2010 research, significant improvements were observed in the SF-36 health survey scores for individuals with knee osteoarthritis following aquatic therapy [31]. Aquatic therapy stands out in pain management, possibly due to the reduced impact and increased comfort of exercising in water compared to land [32].

Both high-energy and low-energy laser therapies exhibit pronounced therapeutic effects for the management of knee osteoarthritis (KOA). This study reveals that high-energy laser therapy, in terms of overall efficacy, is surpassed only by knee orthoses, hydrotherapy, and exercise therapy, with low-energy laser therapy following extracorporeal shockwave therapy. In terms of pain alleviation, the efficacy of both high-energy and low-energy laser therapies is secondary only to that of hydrotherapy. Owing to its non-invasive mechanical action in therapy, low-level laser therapy has emerged as a primary treatment option for a variety of musculoskeletal pain conditions. Clinically, it has been shown to mitigate pain and inflammation, facilitate healing and tissue repair, and enhance blood circulation [33,34]. High-intensity laser therapy, providing concentrated laser energy over a brief period, penetrates deeper into tissues, eliciting a more potent biostimulative and anti-inflammatory response [35]. Ahmad [11] et al., through a systematic review, have demonstrated the superiority of high-intensity laser therapy over low-intensity laser therapy in treating knee osteoarthritis, aligning with the findings of this study.

Exercise plays an extensive role in KOA management. Goh et al. [6] conducted a network meta-analysis to assess the efficacy and safety of various exercises for KOA. The American College of Sports Medicine categorizes exercises into muscle strengthening, aerobic activities, flexibility practices, and neuromuscular training. Substantial evidence supports the improvement of KOA symptoms, including pain, functionality, and quality of life, through exercise intervention [10,36–39]. Exercise modalities for KOA are diverse, with aerobic and mind-body exercises showing the most significant benefits for pain and function, while strengthening and flexibility/skill exercises are the next best options. Mixed exercises are deemed the least effective for knee and hip osteoarthritis [6,40]. A prior network meta-analysis conducted by Mo et al. [41] investigated the clinical efficacy of five different exercise therapies for the management of knee osteoarthritis, including aquatic exercise (AE), stationary cycling (CY), resistance training (RT), traditional exercise (TC), and yoga (YG). The findings concluded that AE (for pain relief) and YG (for alleviating joint stiffness, improving knee function, and enhancing quality of life) are the most effective interventions, followed by RT, CY, and TC. This study considers various exercise therapies as a single intervention for comparison with other treatment modalities, indicating that exercise therapy is effective in improving knee joint function and warrants broader clinical application.

Pulsed ultrasound’s mechanical and thermal effects offer a potential treatment option for mild to moderate KOA, alleviating pain, enhancing mobility, accelerating tissue healing, and reducing edema and disability [42]. However, the role of ultrasound pulse therapy in KOA remains contentious. Previous studies have applied unfocused continuous ultrasound at 1 or 1.5 MHz and 1-2.5 W/cm to the muscles or tendons around the knee [43,44], primarily inducing thermal effects to boost blood circulation and ease muscle and tendon spasms. Some research suggests that this thermal effect may not sufficiently address spasms due to inadequate energy absorption by the muscles, which could account for the temporary pain relief observed with pulsed ultrasound therapy in KOA [45,46]. This study’s conclusions align with these findings, noting that while pulsed ultrasound is moderately effective for pain improvement, it does not significantly enhance other knee joint functions.

Wedge insoles, similar to knee orthoses, primarily aim to alleviate knee joint stress by modifying joint load. Clinically, lateral wedge insoles are predominantly utilized. However, studies indicate that they do not outperform neutral devices in pain reduction. The reduction of the knee adduction moment by only 5%-6% may be insufficient for pain alleviation. Additionally, factors like the sagittal moment and muscle co-contraction might have a more profound impact on the medial knee load, suggesting that a decrease in the adduction moment alone is not adequate to improve KOA function and pain [47–49].

When evaluating overall treatment efficacy, emphasis is placed on the WOMAC score and the resting VAS pain score, as these assessments are nearly universally conducted in the literature, thus offering a more robust framework for both direct and indirect comparative analyses. While other scale assessments are equally important, they lack the same level of comparative data, such as comparisons between the total WOMAC score and the VAS pain score during physical activity.

This study acknowledges several limitations. Firstly, variations in the duration of physical therapy across studies introduce a degree of heterogeneity in the included literature. Secondly, the majority of included studies have small sample sizes, and there are variations in gender distribution. Thirdly, despite the widespread use of WOMAC and VAS scores, there are inconsistencies in other assessment metrics. Fourthly, the methodology of the included studies, particularly regarding randomization and blinding, exhibits certain inadequacies. Lastly, the inclusion of English-language literature only may introduce language bias. Future research should prioritize evaluating the clinical efficacy of combined therapeutic approaches for KOA, as well as their cost-effectiveness and associated health-related expenses.

## 5 Conclusion

In conclusion, the findings suggest that knee brace may be the most recommended therapeutic option for the knee osteoarthritis followed by hydrotherapy and exercise.

## Supporting information

S1 Checklist PRISMA 2020 checklist. (DOCX)

S1 File Minimal data set. (XLS)

S1 Table Searching strategy for PubMed. (DOCX)

S2 Table Table of all data extracted. (DOC)
