# Supplementary material for: Clinical efficacy of different therapeutic options for knee osteoarthritis: A network meta-analysis based on randomized clinical trials
Source: PLoS One. 2025 Jun 18;20(6):e0324864. doi: 10.1371/journal.pone.0324864 (PMC12176148; doi:10.1371/journal.pone.0324864)
Supplement: S1 Table — (DOCX) [file pone.0324864.s003.docx]

**S1 Table. Searching strategy for PubMed.**

| **No.** | **Search items** |
| --- | --- |
| **#**1 | “osteoarthritis”[All Fields] |
| #2 | “knee”[All Fields] |
| #3 = #1 AND #2 | |
| #4 | “**hydrotherapy**”[All Fields] OR “**low level laser therapy** ”[All Fields] OR “**high intensity laser therapy**”[All Fields] OR “**transcutaneous electrical nerve stimulation**”[All Fields] OR “**short wave diathermy**”[All Fields] OR “**interferential current**”[All Fields] OR “**exercise**”[All Fields] OR “**ultrasound**”[All Fields] OR “**brace**”[All Fields] OR “**wedged**”[All Fields] OR “**insole**”[All Fields] OR “**Kinesio Taping**”[All Fields] OR “**tape**”[All Fields] OR “**valgus**”[All Fields] OR “**extracorporeal shock wave therapy**”[All Fields] |
| #5 | “random”[All Fields] |
| #6= #3 AND #4 AND #5 | |
