# Supplementary material for: Clinical efficacy of different therapeutic options for knee osteoarthritis: A network meta-analysis based on randomized clinical trials
Source: PLoS One. 2025 Jun 18;20(6):e0324864. doi: 10.1371/journal.pone.0324864 (PMC12176148; doi:10.1371/journal.pone.0324864)
Supplement: S2 Table — (DOC) [file pone.0324864.s004.doc]

**Number table of all studies identified in the literature search for PubMed**

| **ID** | **Title** | **Doi or PMID** | **Included or Excluded** | **Reason for exclusion** |
| --- | --- | --- | --- | --- |
