# Supplementary material for: Clinical efficacy of different therapeutic options for knee osteoarthritis: A network meta-analysis based on randomized clinical trials
Source: PLoS One. 2025 Jun 18;20(6):e0324864. doi: 10.1371/journal.pone.0324864 (PMC12176148; doi:10.1371/journal.pone.0324864)
Supplement: Attachment — Submitted filename: Response to Reviewer.doc [file pone.0324864.s008.doc]

Dear reviewer and editor,

Firstly, I would like to thank you for your valuable comments on this study. I will answer them one by one and revise them carefully!! And we look forward to your further comments, thanks!!

**General Comments**

**Originality and Relevance:**

Reviewer 1 highlighted the need to address how this review differs from a recent systematic review by Mo et al. (2023). Please include a comparison with this study in the discussion to clarify the manuscript’s originality and contribution.

Answer: This study is distinct from the research by Mo et al. (Mo L, Jiang B, Mei T, Zhou D. Exercise Therapy for Knee Osteoarthritis: A Systematic Review and Network Meta-analysis. Orthop J Sports Med. 2023 Jun 5;11(5):23259671231172773. doi: 10.1177/23259671231172773. PMID: 37346776; PMCID: PMC10280533.) in the following respects: (1)The interventions assessed differ: Mo et al.'s study focused on the clinical efficacy of five exercise therapies for knee osteoarthritis (aquatic exercise [AE], stationary cycling [CY], resistance training [RT], traditional exercise [TC], and yoga [YG]). This study, however, investigates the clinical efficacy of a broader range of non-surgical, non-pharmacological treatment methods for knee osteoarthritis, encompassing 12 intervention measures (low level laser therapy [LLLT], high intensity laser therapy [HILT], transcutaneous electrical nerve stimulation [TENS], interferential current [IFC], short wave diathermy, ultrasound, lateral wedged insole, knee brace, exercise, hydrotherapy, kinesio taping [KT], and extracorporeal shock wave therapy [ESWT]), categorizing various exercise therapies as a single intervention for comparative purposes with other treatment modalities; (2) he study and sample size inclusion differ: Mo et al.'s study included 39 studies with 2646 patients, while this study includes 139 RCTs with 9644 patients, indicating a substantially larger number of studies and a larger sample size, which enhances the persuasiveness of the findings.

I will include a comparison with this study in the discussion to clarify the manuscript’s originality and contribution in red letters in my article.

**Clarity and Focus:**

The aim and title require refinement. Therapies such as laser and ultrasound, which are not strictly physical therapies, should be broadly categorized under "therapeutic options." This will ensure that the scope of the manuscript is accurately reflected.

Answer: Thank you for your valuable advice. I will revise the title and aim from “physical therapies” to “therapeutic options” in red letters in my article.

**Technical Improvements:**

Figures, including funnel plots and forest plots, need to be provided in high resolution to improve readability.

Answer: Thank you for your valuable advice. I will upload clear and higher resolution pictures.

Include a detailed search strategy for at least one database to enhance transparency.

Answer: Thank you for your valuable advice. I will upload a search strategy using the PubMed database as an example. As following:

**Searching strategy for PubMed.**

| **No.** | **Search items** |
| --- | --- |
| **#**1 | “osteoarthritis”[All Fields] |
| #2 | “knee”[All Fields] |
| #3 = #1 AND #2 | |
| #4 | “**hydrotherapy**”[All Fields] OR “**low level laser therapy** ”[All Fields] OR “**high intensity laser therapy**”[All Fields] OR “**transcutaneous electrical nerve stimulation**”[All Fields] OR “**short wave diathermy**”[All Fields] OR “**interferential current**”[All Fields] OR “**exercise**”[All Fields] OR “**ultrasound**”[All Fields] OR “**brace**”[All Fields] OR “**wedged**”[All Fields] OR “**insole**”[All Fields] OR “**Kinesio Taping**”[All Fields] OR “**tape**”[All Fields] OR “**valgus**”[All Fields] OR “**extracorporeal shock wave therapy**”[All Fields] |
| #5 | “random”[All Fields] |
| #6= #3 AND #4 AND #5 | |

**Specific Section-wise Feedback**

Title and Aim

Revise the title and aim to use broader terminology such as “therapeutic options” instead of "physical therapy" to encompass all assessed modalities (Reviewer 1).

Answer: Thank you for your valuable advice. I will revise the title and aim from “physical therapies” to “therapeutic options” in red letters in my article.

Abstract

Clearly mention the scores or metrics used to assess efficacy in the Methods section (Reviewer 1).

Introduction

Provide information on the prevalence of KOA among younger populations in addition to those aged >60 years (Reviewer 1).

Answer: A global study revealed that the global prevalence of knee osteoarthritis (KOA) among individuals aged 15 years and older is 16.0% (95% CI, 14.3%−17.8%), and it escalates to 22.9% (95% CI, 19.8%−26.1%) in those aged 40 years and older. The study also highlighted a significant increase in the incidence of KOA from 1990 to 2019 across the age groups 0-34, 35-39, and 40-44 years. A population-based study reported a global aggregated incidence rate of knee osteoarthritis in individuals aged 20 years and older of 203 cases per 10,000 person-years. This indicates that the incidence of KOA is present in younger age groups (20-59 years), albeit potentially lower than in older demographics. A longitudinal study of 17.7 million Chinese adults from 2008 to 2017 found that the 10-year average age-standardized prevalence and incidence of knee osteoarthritis were 4.6% and 25.2 cases per 1000 person-years, respectively. The study observed an increasing trend in prevalence among individuals under 35 years of age, suggesting a decreasing trend in the age of onset for knee osteoarthritis. A systematic review and meta-analysis indicated that the overall prevalence of knee osteoarthritis in Chinese individuals aged 40 and older was 17.0%, with 12.3% in males and 22.2% in females. Although this study primarily focused on the elderly population aged 40 and above, it also provided data on the prevalence of KOA among younger adults (40-59 years). Another study noted that the prevalence of osteoarthritis increases with age, with 2983.5 per 100,000 cases in the population aged 25-49 and 23,237.2 per 100,000 cases in the population aged 50-69 in 2020. These data further confirm the prevalence of KOA in younger populations and demonstrate an increasing trend in prevalence with age.

Reference:

[1]	Kang Y, Liu C, Ji Y, et al. The burden of knee osteoarthritis worldwide, regionally, and nationally from 1990 to 2019, along with an analysis of cross-national inequalities[J]. Arch Orthop Trauma Surg, 2024, 144(6): 2731-2743.

[2]	Sun W, Yuwen P, Yang X, et al. Changes in epidemiological characteristics of knee arthroplasty in eastern, northern and central China between 2011 and 2020[J]. J Orthop Surg Res, 2023, 18(1): 104.

[3]	Geng R, Li J, Yu C, et al. Knee osteoarthritis: Current status and research progress in treatment (Review)[J]. Exp Ther Med, 2023, 26(4): 481.

[4]	Lv Z, Yang YX, Li J, et al. Molecular Classification of Knee Osteoarthritis[J]. Front Cell Dev Biol, 2021, 9: 725568.

[5]	Chen H, Wu J. Trends and Patterns of Knee Osteoarthritis in China: A Longitudinal Study of 17.7 Million Adults from 2008 to 2017[J]. 2021, 18(16).

Methods

Population Differences:

Reviewer 1 suggests accounting for differences in gender, BMI, and therapy duration in the network meta-analysis. Please address how these variables might influence the results and indicate whether sensitivity analyses were conducted.

Answer:

In network meta-analysis, considerations were given to differences in gender, body mass index (BMI), and treatment duration. These variables can significantly influence outcomes due to their potential impact on disease progression, treatment response, and variability in patient populations. To elucidate their effects, the following explanations are provided: (1)Gender: Differences in gender may affect the prevalence and severity of knee osteoarthritis (KOA) due to anatomical, physiological, and hormonal differences between males and females. For instance, women have a higher prevalence of KOA, which may be attributed to hormonal influences and a higher lifetime risk of joint injury. (2)Body Mass Index (BMI): BMI is a significant factor in KOA as it is directly related to the mechanical stress on weight-bearing joints. Higher BMI increases the risk of developing KOA, and it is also associated with a poorer response to treatment and a higher likelihood of disease progression. (3)Treatment Duration: The duration of treatment can influence the effectiveness of interventions. Longer treatment periods may allow for more significant improvements in symptoms and function, but they may also be associated with increased risks of adverse events or treatment-related complications.

This study performed a sensitivity analysis by sequentially excluding certain studies stratified by gender, BMI, and treatment duration to assess the stability of the results. The findings remained stable after the exclusion of individual studies, demonstrating the robustness of the results for each indicator and indicating that they were not affected by the variability in gender, BMI, or treatment duration.

Search Strategy:

Reviewer 2 highlights the absence of a detailed search strategy. Please attach the search strategies for at least two databases.

Answer:

**(1)Searching strategy for PubMed.**

| **No.** | **Search items** |
| --- | --- |
| **#**1 | “osteoarthritis”[All Fields] |
| #2 | “knee”[All Fields] |
| #3 = #1 AND #2 | |
| #4 | “**hydrotherapy**”[All Fields] OR “**low level laser therapy** ”[All Fields] OR “**high intensity laser therapy**”[All Fields] OR “**transcutaneous electrical nerve stimulation**”[All Fields] OR “**short wave diathermy**”[All Fields] OR “**interferential current**”[All Fields] OR “**exercise**”[All Fields] OR “**ultrasound**”[All Fields] OR “**brace**”[All Fields] OR “**wedged**”[All Fields] OR “**insole**”[All Fields] OR “**Kinesio Taping**”[All Fields] OR “**tape**”[All Fields] OR “**valgus**”[All Fields] OR “**extracorporeal shock wave therapy**”[All Fields] |
| #5 | “random”[All Fields] |
| #6= #3 AND #4 AND #5 | |

**(2) Searching strategy for OVID**

Avoid referring to Google Scholar as "consulted." Instead, clarify that it was "searched" (Reviewer 2).

Answer: Thank you for your valuable advice. I will revise it in red letters in my article.

Protocol Consistency:

Ensure consistent use of terminology regarding meta-analysis, as it is a component of systematic reviews (Reviewer 2).

Answer: Thank you for your valuable advice. I will revise it in red letters in my article.

Results

Subgroup Analysis:

Reviewer 1 inquires about therapy response differences by age group, KOA severity, and comorbidities. If data are available, address these points explicitly.

Answer: This study performed a sensitivity analysis by sequentially excluding certain studies stratified by gender, BMI, treatment duration, KOA severity, and comorbidities to assess the stability of the results. The findings remained stable after the exclusion of individual studies, demonstrating the robustness of the results for each indicator and indicating that they were not affected by the variability in gender, BMI, treatment duration, KOA severity, or comorbidities.

Figures:

Submit high-resolution versions of figures to avoid pixelation (Reviewer 1, Reviewer 2).

Answer: Thank you for your valuable advice. I will upload clear and higher resolution pictures.

Discussion

Comparative Analysis:

Discuss the potential benefits of combination therapies and whether any equipoise exists for future research (Reviewer 1).

Answer: Thank you for your valuable advice. This study focused on the clinical efficacy of monotherapy for Knee Osteoarthritis and did not explore the efficacy of combination therapies. This identifies a significant research opportunity for our team to undertake a comprehensive network meta-analysis in the future, dedicated to elucidating the potential advantages of combined therapeutic approaches.

Contextualization:

Highlight how your findings differ from the systematic review by Mo et al. (2023) to emphasize the manuscript’s contribution.

Answer: This study is distinct from the research by Mo et al. (Mo L, Jiang B, Mei T, Zhou D. Exercise Therapy for Knee Osteoarthritis: A Systematic Review and Network Meta-analysis. Orthop J Sports Med. 2023 Jun 5;11(5):23259671231172773. doi: 10.1177/23259671231172773. PMID: 37346776; PMCID: PMC10280533.) in the following respects: (1)The interventions assessed differ: Mo et al.'s study focused on the clinical efficacy of five exercise therapies for knee osteoarthritis (aquatic exercise [AE], stationary cycling [CY], resistance training [RT], traditional exercise [TC], and yoga [YG]). This study, however, investigates the clinical efficacy of a broader range of non-surgical, non-pharmacological treatment methods for knee osteoarthritis, encompassing 12 intervention measures (low level laser therapy [LLLT], high intensity laser therapy [HILT], transcutaneous electrical nerve stimulation [TENS], interferential current [IFC], short wave diathermy, ultrasound, lateral wedged insole, knee brace, exercise, hydrotherapy, kinesio taping [KT], and extracorporeal shock wave therapy [ESWT]), categorizing various exercise therapies as a single intervention for comparative purposes with other treatment modalities; (2)The study and sample size inclusion differ: Mo et al.'s study included 39 studies with 2646 patients, while this study includes 139 RCTs with 9644 patients, indicating a substantially larger number of studies and a larger sample size, which enhances the persuasiveness of the findings.

Formatting and Referencing

Write all numbers below 10 in words (Reviewer 2).

Adhere to the journal’s referencing style guidelines, which require DOI but not PMID (Reviewer 2).

Answer: Thank you for your valuable advice. I will revise it in red letters in my article.

Additional Comments from Reviewers

Clearly define the review question (Reviewer 2).

Use AMSTAR appropriately as a critical appraisal tool for systematic reviews (Reviewer 2).

**Conclusion**

The manuscript is comprehensive and addresses a significant clinical question. However, revisions are necessary to enhance its originality, methodological clarity, and alignment with journal standards. Once these revisions are implemented, the manuscript will be well-positioned for further consideration.

Answer: Thank you for your valuable advice.

**We look forward to receiving your revised submission.**

Please submit your revised manuscript by Jan 31 2025 11:59PM. If you will need more time than this to complete your revisions, please reply to this message or contact the journal office at [plosone@plos.org](mailto:plosone@plos.org). When you're ready to submit your revision, log on to <https://www.editorialmanager.com/pone/> and select the 'Submissions Needing Revision' folder to locate your manuscript file.

Please include the following items when submitting your revised manuscript:

- A rebuttal letter that responds to each point raised by the academic editor and reviewer(s). You should upload this letter as a separate file labeled 'Response to Reviewers'.

Answer: I will upload it.

- A marked-up copy of your manuscript that highlights changes made to the original version. You should upload this as a separate file labeled 'Revised Manuscript with Track Changes'.

Answer: I will upload it.

- An unmarked version of your revised paper without tracked changes. You should upload this as a separate file labeled 'Manuscript'.

Answer: I will upload it.

If you would like to make changes to your financial disclosure, please include your updated statement in your cover letter. Guidelines for resubmitting your figure files are available below the reviewer comments at the end of this letter.

If applicable, we recommend that you deposit your laboratory protocols in protocols.io to enhance the reproducibility of your results. Protocols.io assigns your protocol its own identifier (DOI) so that it can be cited independently in the future. For instructions see: [https://journals.plos.org/plosone/s/submission-guidelines#loc-laboratory-protocols](https://track.editorialmanager.com/CL0/https://journals.plos.org/plosone/s/submission-guidelines%23loc-laboratory-protocols/1/010f0193d320a6f8-9c7f751a-28c3-4b2f-b4ce-5dc3e130460f-000000/LQoMmhchN6TTUXeYJsueGmr4r-P3Xgpg4IVScRgvIoE=189). Additionally, PLOS ONE offers an option for publishing peer-reviewed Lab Protocol articles, which describe protocols hosted on protocols.io. Read more information on sharing protocols at [https://plos.org/protocols?utm_medium=editorial-email&utm_source=authorletters&utm_campaign=protocols](https://track.editorialmanager.com/CL0/https://plos.org/protocols?utm_medium=editorial-email&utm_source=authorletters&utm_campaign=protocols/1/010f0193d320a6f8-9c7f751a-28c3-4b2f-b4ce-5dc3e130460f-000000/vbGRVij9ZWTj5lhsweFkggRWJeoxs9won1UUSZ6ZH8I=189).

We look forward to receiving your revised manuscript.

Kind regards,

Clementswami Sukumaran, PhD
Academic Editor
PLOS ONE

**Journal Requirements:**

1. When submitting your revision, we need you to address these additional requirements.

Please ensure that your manuscript meets PLOS ONE's style requirements, including those for file naming. The PLOS ONE style templates can be found at
<https://journals.plos.org/plosone/s/file?id=wjVg/PLOSOne_formatting_sample_main_body.pdf> and
<https://journals.plos.org/plosone/s/file?id=ba62/PLOSOne_formatting_sample_title_authors_affiliations.pdf>

2. We note that the grant information you provided in the ‘Funding Information’ and ‘Financial Disclosure’ sections do not match.

When you resubmit, please ensure that you provide the correct grant numbers for the awards you received for your study in the ‘Funding Information’ section.

3. PLOS requires an ORCID iD for the corresponding author in Editorial Manager on papers submitted after December 6th, 2016. Please ensure that you have an ORCID iD and that it is validated in Editorial Manager. To do this, go to ‘Update my Information’ (in the upper left-hand corner of the main menu), and click on the Fetch/Validate link next to the ORCID field. This will take you to the ORCID site and allow you to create a new iD or authenticate a pre-existing iD in Editorial Manager.

4. Please upload a new copy of Figures 1, 3, 4, 5 and 6 as the detail is not clear. Please follow the link for more information: <https://blogs.plos.org/plos/2019/06/looking-good-tips-for-creating-your-plos-figures-graphics/>" <https://blogs.plos.org/plos/2019/06/looking-good-tips-for-creating-your-plos-figures-graphics/>"

Answer: I will upload it.

5. As required by our policy on Data Availability, please ensure your manuscript or supplementary information includes the following:

A numbered table of all studies identified in the literature search, including those that were excluded from the analyses.

For every excluded study, the table should list the reason(s) for exclusion.

If any of the included studies are unpublished, include a link (URL) to the primary source or detailed information about how the content can be accessed.

A table of all data extracted from the primary research sources for the systematic review and/or meta-analysis. The table must include the following information for each study:

Name of data extractors and date of data extraction

Confirmation that the study was eligible to be included in the review.

All data extracted from each study for the reported systematic review and/or meta-analysis that would be needed to replicate your analyses.

If data or supporting information were obtained from another source (e.g. correspondence with the author of the original research article), please provide the source of data and dates on which the data/information were obtained by your research group.

If applicable for your analysis, a table showing the completed risk of bias and quality/certainty assessments for each study or outcome.  Please ensure this is provided for each domain or parameter assessed. For example, if you used the Cochrane risk-of-bias tool for randomized trials, provide answers to each of the signalling questions for each study. If you used GRADE to assess certainty of evidence, provide judgements about each of the quality of evidence factor. This should be provided for each outcome.

An explanation of how missing data were handled.

This information can be included in the main text, supplementary information, or relevant data repository. Please note that providing these underlying data is a requirement for publication in this journal, and if these data are not provided your manuscript might be rejected.

Answer: I will upload it.

6. Please review your reference list to ensure that it is complete and correct. If you have cited papers that have been retracted, please include the rationale for doing so in the manuscript text, or remove these references and replace them with relevant current references. Any changes to the reference list should be mentioned in the rebuttal letter that accompanies your revised manuscript. If you need to cite a retracted article, indicate the article’s retracted status in the References list and also include a citation and full reference for the retraction notice.

Answer: Thank you for your valuable advice. I will revise it in red letters in my article.

[Note: HTML markup is below. Please do not edit.]

Reviewers' comments:

Reviewer's Responses to Questions

**Comments to the Author**

1. Is the manuscript technically sound, and do the data support the conclusions?

The manuscript must describe a technically sound piece of scientific research with data that supports the conclusions. Experiments must have been conducted rigorously, with appropriate controls, replication, and sample sizes. The conclusions must be drawn appropriately based on the data presented.

Reviewer #1: Yes

Reviewer #2: Partly

2. Has the statistical analysis been performed appropriately and rigorously?

Reviewer #1: Yes

Reviewer #2: I Don't Know

3. Have the authors made all data underlying the findings in their manuscript fully available?

The [PLOS Data policy](http://track.editorialmanager.com/CL0/http://www.plosone.org/static/policies.action%23sharing/1/010f0193d320a6f8-9c7f751a-28c3-4b2f-b4ce-5dc3e130460f-000000/7goJl8MAdHd-4fw9JESBKBUBNTC0SjASnJCLBMzglFM=189) requires authors to make all data underlying the findings described in their manuscript fully available without restriction, with rare exception (please refer to the Data Availability Statement in the manuscript PDF file). The data should be provided as part of the manuscript or its supporting information, or deposited to a public repository. For example, in addition to summary statistics, the data points behind means, medians and variance measures should be available. If there are restrictions on publicly sharing data—e.g. participant privacy or use of data from a third party—those must be specified.

Reviewer #1: Yes

Reviewer #2: Yes

4. Is the manuscript presented in an intelligible fashion and written in standard English?

PLOS ONE does not copyedit accepted manuscripts, so the language in submitted articles must be clear, correct, and unambiguous. Any typographical or grammatical errors should be corrected at revision, so please note any specific errors here.

Reviewer #1: Yes

Reviewer #2: Yes

5. Review Comments to the Author

Please use the space provided to explain your answers to the questions above. You may also include additional comments for the author, including concerns about dual publication, research ethics, or publication ethics. (Please upload your review as an attachment if it exceeds 20,000 characters)

**Reviewer #1:** I have reviewed the manuscript titles: “Clinical efficacy of different physical therapies for knee osteoarthritis: a network meta-analysis based on randomized clinical trials” with great interest.
This is a very well written review and meta-analysis
There are several questions and points that I highlighted in the attached word document..

Answer: Thank you for your valuable advice. I will revise it according to your questions and points.

**Reviewer #2:** The authors have presented a comprehensive analysis of the clinical efficacy of various physical therapies in treating knee osteoarthritis (KOA).
Though the authors have mentioned the objective, the review question has not been mentioned.
Write all numbers below 10 in words
The protocol and the review is different, including the title.
AMSTAR is a critical appraisal tool for systematic review.
Google scholar is a grey literature database. Google scholar cannot be consulted, but searched
The authors have mentioned meta analysis in the exclusion criteria. Meta analysis is the analysis done in a systematic review, it is a part of systematic review.
Read the guidelines to authors for referencing style. PMID is not required. only DOI is required
The funnel plots and forest plots are too small and cannot be read
A search strategy of at least one database should be provided.

Answer: Thank you for your valuable advice. I will revise it according to your questions.

6. PLOS authors have the option to publish the peer review history of their article ([what does this mean?](https://track.editorialmanager.com/CL0/https://journals.plos.org/plosone/s/editorial-and-peer-review-process%23loc-peer-review-history/1/010f0193d320a6f8-9c7f751a-28c3-4b2f-b4ce-5dc3e130460f-000000/HBwtMwptAHRGycaBugJEus3Z9SNxjD_rV8irrFIbMd4=189)). If published, this will include your full peer review and any attached files.


If you choose “no”, your identity will remain anonymous but your review may still be made public.


**Do you want your identity to be public for this peer review?** For information about this choice, including consent withdrawal, please see our [Privacy Policy](https://track.editorialmanager.com/CL0/https://www.plos.org/privacy-policy/1/010f0193d320a6f8-9c7f751a-28c3-4b2f-b4ce-5dc3e130460f-000000/8v2SllJpeS7MLJshL2o-1hT2HLUKyKT_1bZUL6RnG74=189).

Reviewer #1: **Yes:** Noora Alshahwani

Reviewer #2: No

**Reviewer #1:**

I have reviewed the manuscript titles: “Clinical efficacy of different physical therapies for knee osteoarthritis: a network meta-analysis based on randomized clinical trials” with great interest.

This is a very well written review and meta-analysis

Below are my comments for each sections:

Originality: There is a recent systematic review published in 2023 looking at effects of physiotherapy in knee osteoarthritis (cited below). How is this review different from that one? It would be relevant to include that one in the discussion and highlight how yours is different.

Mo L, Jiang B, Mei T, Zhou D. Exercise Therapy for Knee Osteoarthritis: A Systematic Review and Network Meta-analysis. Orthop J Sports Med. 2023 Jun 5;11(5):23259671231172773. doi: 10.1177/23259671231172773. PMID: 37346776; PMCID: PMC10280533.

Answer: This study is distinct from the research by Mo et al. (Mo L, Jiang B, Mei T, Zhou D. Exercise Therapy for Knee Osteoarthritis: A Systematic Review and Network Meta-analysis. Orthop J Sports Med. 2023 Jun 5;11(5):23259671231172773. doi: 10.1177/23259671231172773. PMID: 37346776; PMCID: PMC10280533.) in the following respects: (1)The interventions assessed differ: Mo et al.'s study focused on the clinical efficacy of five exercise therapies for knee osteoarthritis (aquatic exercise [AE], stationary cycling [CY], resistance training [RT], traditional exercise [TC], and yoga [YG]). This study, however, investigates the clinical efficacy of a broader range of non-surgical, non-pharmacological treatment methods for knee osteoarthritis, encompassing 12 intervention measures (low level laser therapy [LLLT], high intensity laser therapy [HILT], transcutaneous electrical nerve stimulation [TENS], interferential current [IFC], short wave diathermy, ultrasound, lateral wedged insole, knee brace, exercise, hydrotherapy, kinesio taping [KT], and extracorporeal shock wave therapy [ESWT]), categorizing various exercise therapies as a single intervention for comparative purposes with other treatment modalities; (2) The study and sample size inclusion differ: Mo et al.'s study included 39 studies with 2646 patients, while this study includes 139 RCTs with 9644 patients, indicating a substantially larger number of studies and a larger sample size, which enhances the persuasiveness of the findings.

I will include a comparison with this study in the discussion to clarify the manuscript’s originality and contribution in red letters in my article.

By section:

Title and Aim: The aim is to assess the efficacy of various physical therapy options. Laser and ultrasound therapies are considered therapies that complement physical therapy, yet aren’t classified under physical therapy. Consider changing the aim, title and any other statement to make it more general “e.g. therapeutic options” to encompass all assessed modalities.

Answer: Thank you for your valuable advice. I will revise the title and aim from “physical therapies” to “therapeutic options” in red letters in my article.

Abstract: In methods mention the scores used to assess efficacy of the different therapies.

Thank you for your valuable advice. I will revise it in red letters in my article and highlight it with yellow.

Introduction: You provided a statement highlighting the occurrence of KOA in elderly >60 years old. How often is it seen in the younger population?

Answer: A global study revealed that the global prevalence of knee osteoarthritis (KOA) among individuals aged 15 years and older is 16.0% (95% CI, 14.3%−17.8%), and it escalates to 22.9% (95% CI, 19.8%−26.1%) in those aged 40 years and older. The study also highlighted a significant increase in the incidence of KOA from 1990 to 2019 across the age groups 0-34, 35-39, and 40-44 years. A population-based study reported a global aggregated incidence rate of knee osteoarthritis in individuals aged 20 years and older of 203 cases per 10,000 person-years. This indicates that the incidence of KOA is present in younger age groups (20-59 years), albeit potentially lower than in older demographics. A longitudinal study of 17.7 million Chinese adults from 2008 to 2017 found that the 10-year average age-standardized prevalence and incidence of knee osteoarthritis were 4.6% and 25.2 cases per 1000 person-years, respectively. The study observed an increasing trend in prevalence among individuals under 35 years of age, suggesting a decreasing trend in the age of onset for knee osteoarthritis. A systematic review and meta-analysis indicated that the overall prevalence of knee osteoarthritis in Chinese individuals aged 40 and older was 17.0%, with 12.3% in males and 22.2% in females. Although this study primarily focused on the elderly population aged 40 and above, it also provided data on the prevalence of KOA among younger adults (40-59 years). Another study noted that the prevalence of osteoarthritis increases with age, with 2983.5 per 100,000 cases in the population aged 25-49 and 23,237.2 per 100,000 cases in the population aged 50-69 in 2020. These data further confirm the prevalence of KOA in younger populations and demonstrate an increasing trend in prevalence with age.

Reference:

[1]	Kang Y, Liu C, Ji Y, et al. The burden of knee osteoarthritis worldwide, regionally, and nationally from 1990 to 2019, along with an analysis of cross-national inequalities[J]. Arch Orthop Trauma Surg, 2024, 144(6): 2731-2743.

[2]	Sun W, Yuwen P, Yang X, et al. Changes in epidemiological characteristics of knee arthroplasty in eastern, northern and central China between 2011 and 2020[J]. J Orthop Surg Res, 2023, 18(1): 104.

[3]	Geng R, Li J, Yu C, et al. Knee osteoarthritis: Current status and research progress in treatment (Review)[J]. Exp Ther Med, 2023, 26(4): 481.

[4]	Lv Z, Yang YX, Li J, et al. Molecular Classification of Knee Osteoarthritis[J]. Front Cell Dev Biol, 2021, 9: 725568.

[5]	Chen H, Wu J. Trends and Patterns of Knee Osteoarthritis in China: A Longitudinal Study of 17.7 Million Adults from 2008 to 2017[J]. 2021, 18(16).

Methods:

- How did you account for the differences in the populations gender, BMI, and duration of therapy in the network meta-analysis? What effect do you anticipate that to have on your results? Where there any sensitivity studies done to check for the effects?

Answer:

In network meta-analysis, considerations were given to differences in gender, body mass index (BMI), and treatment duration. These variables can significantly influence outcomes due to their potential impact on disease progression, treatment response, and variability in patient populations. To elucidate their effects, the following explanations are provided: (1)Gender: Differences in gender may affect the prevalence and severity of knee osteoarthritis (KOA) due to anatomical, physiological, and hormonal differences between males and females. For instance, women have a higher prevalence of KOA, which may be attributed to hormonal influences and a higher lifetime risk of joint injury. (2)Body Mass Index (BMI): BMI is a significant factor in KOA as it is directly related to the mechanical stress on weight-bearing joints. Higher BMI increases the risk of developing KOA, and it is also associated with a poorer response to treatment and a higher likelihood of disease progression. (3)Treatment Duration: The duration of treatment can influence the effectiveness of interventions. Longer treatment periods may allow for more significant improvements in symptoms and function, but they may also be associated with increased risks of adverse events or treatment-related complications.

This study performed a sensitivity analysis by sequentially excluding certain studies stratified by gender, BMI, and treatment duration to assess the stability of the results. The findings remained stable after the exclusion of individual studies, demonstrating the robustness of the results for each indicator and indicating that they were not affected by the variability in gender, BMI, or treatment duration.

- I suggest you attach your exact search strategy if feasible per database (include atleast 2).

Answer:

**(1)Searching strategy for PubMed.**

| **No.** | **Search items** |
| --- | --- |
| **#**1 | “osteoarthritis”[All Fields] |
| #2 | “knee”[All Fields] |
| #3 = #1 AND #2 | |
| #4 | “**hydrotherapy**”[All Fields] OR “**low level laser therapy** ”[All Fields] OR “**high intensity laser therapy**”[All Fields] OR “**transcutaneous electrical nerve stimulation**”[All Fields] OR “**short wave diathermy**”[All Fields] OR “**interferential current**”[All Fields] OR “**exercise**”[All Fields] OR “**ultrasound**”[All Fields] OR “**brace**”[All Fields] OR “**wedged**”[All Fields] OR “**insole**”[All Fields] OR “**Kinesio Taping**”[All Fields] OR “**tape**”[All Fields] OR “**valgus**”[All Fields] OR “**extracorporeal shock wave therapy**”[All Fields] |
| #5 | “random”[All Fields] |
| #6= #3 AND #4 AND #5 | |

**(2) Searching strategy for OVID**

Results:

- Is there a difference in the response to therapy according to age group? Severity of KOA? Comorbidities?

Answer: This study performed a sensitivity analysis by sequentially excluding certain studies stratified by gender, BMI, treatment duration, KOA severity, and comorbidities to assess the stability of the results. The findings remained stable after the exclusion of individual studies, demonstrating the robustness of the results for each indicator and indicating that they were not affected by the variability in gender, BMI, treatment duration, KOA severity, or comorbidities.

- Figures: could you please provide copies of figures with higher definition, to avoid a pixilated picture?

Answer: Thank you for your valuable advice. I will upload clear and higher resolution pictures.

Discussion:

- You mentioned several therapies. Were there any data addressing the benefit of combination therapies? Is there equipoise in that area to highlight for future studies?

Answer: Thank you for your valuable advice. This study focused on the clinical efficacy of monotherapy for Knee Osteoarthritis and did not explore the efficacy of combination therapies. This identifies a significant research opportunity for our team to undertake a comprehensive network meta-analysis in the future, dedicated to elucidating the potential advantages of combined therapeutic approaches.
