# Supplementary material for: Clinical efficacy of different therapeutic options for knee osteoarthritis: A network meta-analysis based on randomized clinical trials
Source: PLoS One. 2025 Jun 18;20(6):e0324864. doi: 10.1371/journal.pone.0324864 (PMC12176148; doi:10.1371/journal.pone.0324864)
Supplement: Attachment — Submitted filename: PONE-D-24-40762R1.docx [file pone.0324864.s007.docx]

Thank you for the invite to review the second version of the systematic review titled: “

Below are my comments and recommendations per section:

- Throughout: check spacing between words and punctuation. Example: Methods: Details regarding the therapeutic options(intervention and comparison).

Abstract:

- Under results: “Regarding the WOMAC pain score, knee brace was determined to be the most likely to yield the best results, followed by exercise, HILT, ESWT, hydrotherapy, LLLT, KT, TENS, short wave diathermy, IFC, lateral wedged insole, placebo and ultrasound.” - It would be easier for the reader and more pertinent to list top 2-3 intervention, and perhaps lowest intervention, rather than listing all 12. This could be done across all scoring schemes
- Conclusion: The findings suggest that knee brace may be the most recommended therapeutic options for the knee osteoarthritis. - I would be interested to know top 2-3 and worst intervention in the conclusion. You could also add a statement about future assessment of complementary use of several interventions

Introduction:

- “The findings aim to equip clinical practice with the most robust evidence-based guidance.” - this statement is misleading, since there is no guideline development is employed in this review

Methods

- Quality assessment: reduce redundancy in the following paragraph: “For RCTs, the Cochrane Risk of Bias Tool was employed to assess the quality. The risk of bias for the included trials was evaluated by two researchers (the first and second authors) based on the Cochrane Handbook criteria. The criteria covered randomization, allocation concealment, blinding of participants and personnel, blinding of outcome assessors, completeness of outcome data, selective reporting, and other biases. Each domain was classified as having an unclear risk, low risk, or high risk of bias.”

Results:

- “To eliminate duplicate entries, the "Find duplicates" function in EndNote software was employed, resulting in the removal of 531 duplicate studies. Following a thorough screening of titles and abstracts, 2598 irrelevant references were excluded. Subsequently, full-texts were retrieved for the remaining 295 references. - I suggest eliminating this statement above, and replacing with “after duplicates were removed…”. You can refer to the prisma chart for details.
- Evidence network: This was explained neatly. However, results need to be listed here in sentence format in a way that explains the figures and can replace them.

Discussion:

- Discuss whether there is any known benefit to combining several modalities? Is this potentially a future direction you would be exploring?
- What other future studies can this review be used as basis?

Conclusion: Need to improve on the conclusion as advised above in the abstract. This should reflect the studies’ outcomes as well as any other relevant study (e.g. Mo et. al meta-analysis)
