# Supplementary material for: Clinical efficacy of different therapeutic options for knee osteoarthritis: A network meta-analysis based on randomized clinical trials
Source: PLoS One. 2025 Jun 18;20(6):e0324864. doi: 10.1371/journal.pone.0324864 (PMC12176148; doi:10.1371/journal.pone.0324864)
Supplement: Attachment — Submitted filename: Response to Reviewer secondly.doc [file pone.0324864.s010.doc]

Dear reviewer and editor,

Firstly, I would like to thank you for your valuable comments on this study. I will answer them one by one and revise them carefully!! And we look forward to your further comments, thanks!!

The search strategy for PUBMED results are 232 as January 23, 2025. In PRIMSA flowchart, PUBMED search results are written as 2252.

Answer：I search search strategy for PUBMED results again are 2570 as February 21 2025, It shouldn't just be 232 articles. Therefore, the PUBMED results of 2252 articles when I searched was correct.

Thank you for the invite to review the second version of the systematic review titled: “

Below are my comments and recommendations per section:

- Throughout: check spacing between words and punctuation. Example: Methods: Details regarding the therapeutic options(intervention and comparison).

Answer: Thank you for your valuable advice. I will revise it in my article.

Abstract:

- Under results: “Regarding the WOMAC pain score, knee brace was determined to be the most likely to yield the best results, followed by exercise, HILT, ESWT, hydrotherapy, LLLT, KT, TENS, short wave diathermy, IFC, lateral wedged insole, placebo and ultrasound.” - It would be easier for the reader and more pertinent to list top 2-3 intervention, and perhaps lowest intervention, rather than listing all 12. This could be done across all scoring schemes

Answer: Thank you for your valuable advice. I will revise it in my article.

- Conclusion: The findings suggest that knee brace may be the most recommended therapeutic options for the knee osteoarthritis. - I would be interested to know top 2-3 and worst intervention in the conclusion. You could also add a statement about future assessment of complementary use of several interventions

Answer: Thank you for your valuable advice. I will revise it in my article.

Introduction:

- “The findings aim to equip clinical practice with the most robust evidence-based guidance.” - this statement is misleading, since there is no guideline development is employed in this review

Answer: Thank you for your valuable advice. I will revise it in my article.

Methods

- Quality assessment: reduce redundancy in the following paragraph: “For RCTs, the Cochrane Risk of Bias Tool was employed to assess the quality. The risk of bias for the included trials was evaluated by two researchers (the first and second authors) based on the Cochrane Handbook criteria. The criteria covered randomization, allocation concealment, blinding of participants and personnel, blinding of outcome assessors, completeness of outcome data, selective reporting, and other biases. Each domain was classified as having an unclear risk, low risk, or high risk of bias.”

Answer: Thank you for your valuable advice. I will revise it in my article.

Results:

- “To eliminate duplicate entries, the "Find duplicates" function in EndNote software was employed, resulting in the removal of 531 duplicate studies. Following a thorough screening of titles and abstracts, 2598 irrelevant references were excluded. Subsequently, full-texts were retrieved for the remaining 295 references. - I suggest eliminating this statement above, and replacing with “after duplicates were removed…”. You can refer to the prisma chart for details.

Answer: Thank you for your valuable advice. I will revise it in my article.

- Evidence network: This was explained neatly. However, results need to be listed here in sentence format in a way that explains the figures and can replace them.

Answer: Thank you for your valuable advice.

Discussion:

- Discuss whether there is any known benefit to combining several modalities? Is this potentially a future direction you would be exploring?
- Answer: At present, there is no research on the combination of several treatment modalities, which is the direction our team will explore in the future.
- What other future studies can this review be used as basis?
- Answer: Yes.

Conclusion: Need to improve on the conclusion as advised above in the abstract. This should reflect the studies’ outcomes as well as any other relevant study (e.g. Mo et. al meta-analysis)

Answer: Thank you for your valuable advice. I will revise it in my article.
