# Supplementary material for: Clinical efficacy of different therapeutic options for knee osteoarthritis: A network meta-analysis based on randomized clinical trials
Source: PLoS One. 2025 Jun 18;20(6):e0324864. doi: 10.1371/journal.pone.0324864 (PMC12176148; doi:10.1371/journal.pone.0324864)
Supplement: Attachment — Submitted filename: PONE-D-24-40762R2.docx [file pone.0324864.s009.docx]

Thank you for the invite to review the third version of the systematic review

Below are my comments and recommendations per section:

Funding: “Funding acquisition: Xiao Chen.” was mentioned, with no further details. This is contradicted with the author’s disclosure of no funding received. Please clarify and ensure consistency.

Abstract:

- Conclusion: “placebo was the worst option.” This statement has no value. Please omit.

Methods

- Under statistical analysis: “Within the "Results" section, …” This can be omitted

Results:

- I still don’t see a paragraph explaining the PICO of the included studies in sentence format. I think it is a necessary addition to explain and expand of what table 1 shows.

Discussion:

- Future directions: in addition to combining modalities, one could look at cost-effectiveness or health related costs.

Conclusion: Need to expand on the main conclusion: “In conclusion, the findings suggest that knee brace may be the most recommended therapeutic option for the knee osteoarthritis followed by hydrotherapy and exercise, placebo was the worst option”. As suggested above, there is no need to include placebo in the list.
