# Supplementary material for: Clinical efficacy of different therapeutic options for knee osteoarthritis: A network meta-analysis based on randomized clinical trials
Source: PLoS One. 2025 Jun 18;20(6):e0324864. doi: 10.1371/journal.pone.0324864 (PMC12176148; doi:10.1371/journal.pone.0324864)
Supplement: Attachment — Submitted filename: Response to Reviewer again.doc [file pone.0324864.s011.doc]

Dear reviewer and editor,

Firstly, I would like to thank you for your valuable comments on this study. I will answer them one by one and revise them carefully!! And we look forward to your further comments, thanks!!

Thank you for the invite to review the third version of the systematic review

Below are my comments and recommendations per section:

Funding: “Funding acquisition: Xiao Chen.” was mentioned, with no further details. This is contradicted with the author’s disclosure of no funding received. Please clarify and ensure consistency.

Answer: Thank you for your valuable advice. I will revise it in my article.

Abstract:

- Conclusion: “placebo was the worst option.” This statement has no value. Please omit.

Answer: Thank you for your valuable advice. I will omit it.

Methods

- Under statistical analysis: “Within the "Results" section, …” This can be omitted

Answer: Thank you for your valuable advice. I will eliminate it.

Results:

- I still don’t see a paragraph explaining the PICO of the included studies in sentence format. I think it is a necessary addition to explain and expand of what table 1 shows.

Answer: Thank you for your valuable advice. I will add it in my article.

Discussion:

- Future directions: in addition to combining modalities, one could look at cost-effectiveness or health related costs.

Answer: Thank you for your valuable advice. I will add it in my article.

Conclusion: Need to expand on the main conclusion: “In conclusion, the findings suggest that knee brace may be the most recommended therapeutic option for the knee osteoarthritis followed by hydrotherapy and exercise, placebo was the worst option”. As suggested above, there is no need to include placebo in the list.

Answer: Thank you for your valuable advice. I will remove it from the conclusion.
