# Supplementary material for: Clinical efficacy of different therapeutic options for knee osteoarthritis: A network meta-analysis based on randomized clinical trials
Source: PLoS One. 2025 Jun 18;20(6):e0324864. doi: 10.1371/journal.pone.0324864 (PMC12176148; doi:10.1371/journal.pone.0324864)
Supplement: Attachment — Submitted filename: PONE-D-24-40762 - reviewer comments.docx [file pone.0324864.s005.docx]

I have reviewed the manuscript titles: “Clinical efficacy of different physical therapies for knee osteoarthritis: a network meta-analysis based on randomized clinical trials” with great interest.

This is a very well written review and meta-analysis

Below are my comments for each sections:

Originality: There is a recent systematic review published in 2023 looking at effects of physiotherapy in knee osteoarthritis (cited below). How is this review different from that one? It would be relevant to include that one in the discussion and highlight how yours is different.

Mo L, Jiang B, Mei T, Zhou D. Exercise Therapy for Knee Osteoarthritis: A Systematic Review and Network Meta-analysis. Orthop J Sports Med. 2023 Jun 5;11(5):23259671231172773. doi: 10.1177/23259671231172773. PMID: 37346776; PMCID: PMC10280533.

By section:

Title and Aim: The aim is to assess the efficacy of various physical therapy options. Laser and ultrasound therapies are considered therapies that complement physical therapy, yet aren’t classified under physical therapy. Consider changing the aim, title and any other statement to make it more general “e.g. therapeutic options” to encompass all assessed modalities.

Abstract: In methods mention the scores used to assess efficacy of the different therapies.

Introduction: You provided a statement highlighting the occurrence of KOA in elderly >60 years old. How often is it seen in the younger population?

Methods:

- How did you account for the differences in the populations gender, BMI, and duration of therapy in the network meta-analysis? What effect do you anticipate that to have on your results? Where there any sensitivity studies done to check for the effects?
- I suggest you attach your exact search strategy if feasible per database (include atleast 2).

Results:

- Is there a difference in the response to therapy according to age group? Severity of KOA? Comorbidities?
- Figures: could you please provide copies of figures with higher definition, to avoid a pixilated picture?

Discussion:

- You mentioned several therapies. Were there any data addressing the benefit of combination therapies? Is there equipoise in that area to highlight for future studies?
